# SCF^AtPP2-B11^ modulates ABA signaling by facilitating SnRK2.3 degradation in *Arabidopsis thaliana*

**DOI:** 10.1371/journal.pgen.1006947

**Published:** 2017-08-07

**Authors:** Chunhong Cheng, Zhijuan Wang, Ziyin Ren, Liya Zhi, Bin Yao, Chao Su, Liu Liu, Xia Li

**Affiliations:** 1 State Key Laboratory of Agricultural Microbiology, College of Plant Science and Technology, Huazhong Agricultural University, Wuhan, P.R., China; 2 Center for Agricultural Resources Research, Institute of Genetics and Developmental Biology, Chinese Academy of Sciences, Shijiazhuang, Hebei, P.R., China; 3 University of Chinese Academy of Sciences, Beijing, P.R., China; Wake Forest University, UNITED STATES

## Abstract

The phytohormone abscisic acid (ABA) is an essential part of the plant response to abiotic stressors such as drought. Upon the perception of ABA, pyrabactin resistance (PYR)/PYR1-like (PYL)/regulatory components of ABA receptor (RCAR) proteins interact with co-receptor protein phosphatase type 2Cs to permit activation Snf1-related protein kinase2 (SnRK2) kinases, which switch on ABA signaling by phosphorylating various target proteins. Thus, SnRK2 kinases are central regulators of ABA signaling. However, the mechanisms that regulate SnRK2 degradation remain elusive. Here, we show that SnRK2.3 is degradated by 26S proteasome system and ABA promotes its degradation. We found that SnRK2.3 interacts with AtPP2-B11 directly. AtPP2-B11 is an F-box protein that is part of a SKP1/Cullin/F-box E3 ubiquitin ligase complex that negatively regulates plant responses to ABA by specifically promoting the degradation of SnRK2.3. *AtPP2-B11* was induced by ABA, and the knockdown of *AtPP2-B11* expression markedly increased the ABA sensitivity of plants during seed germination and postgerminative development. Overexpression of *AtPP2-B11* does not affect ABA sensitivity, but inhibits the ABA hypersensitive phenotypes of *SnRK2*.*3* overexpression lines. These results reveal a novel mechanism through which AtPP2-B11 specifically degrades SnRK2.3 to attenuate ABA signaling and the abiotic stress response in *Arabidopsis*.

## Author summary

The phytohormone ABA plays critical roles in both plant growth and development and the response to environmental stimuli. The SnRK2s are important components of the PYR/PYL-ABA-PP2C regulatory machine which activate a set of bZIP transcription factors and ion transporters. However, the regulation of SnRK2 turnover is poorly understood. In this work, we show that SnRK2s are degraded by 26S proteasome and ABA promotes SnRK2.3 degradation. We also show that an F-box protein, AtPP2-B11, interacts with *Arabidopsis* Skp1-like protein ASK1 and ASK2, is a component of a SKP1/Cullin/F-box E3 ubiquitin ligase complex. AtPP2-B11 interacts and regulates the degradation of SnRK2.3 specifically. *AtPP2-B11* is induced by ABA and knockdown of *AtPP2-B11* expression markedly increases ABA sensitivity of plants during seed germination and post-germinative development. Overexpression of *AtPP2-B11* does not affect ABA sensitivity, but inhibits the ABA hypersensitive phenotypes of *SnRK2*.*3* overexpression lines. These results reveal a mechanism through which AtPP2-B11 specifically degrades SnRK2.3 to attenuate the ABA signaling and plant responses to abiotic stress.

## Introduction

Abscisic acid (ABA) plays important roles in plant development and growth, including seed dormancy, seed germination, and postgerminative growth [1, 2]. In particular, it plays an essential role in plant responses to adverse environmental stresses such as drought and salt stress [1, 2]. The discovery of the pyrabactin resistance (PYR)/PYR1-like (PYL)/regulatory components of ABA receptor (RCAR) protein family, which is comprised of ABA receptors, and intensive research into how these ABA receptors regulate plant responses to ABA have revealed much about the core ABA signaling pathway. This core pathway is mainly composed of PYR/PYL/RCAR ABA receptors, their co-receptors (clade A phosphatase type 2Cs [PP2Cs]), and Snf1-related protein kinase2 (SnRK2) kinases, which phosphorylate bZIP transcription factors and ion channels to turn on ABA signaling [3–5]. In the absence of ABA, PP2Cs interact with SnRK2s and inhibit their kinase activity, turning ABA signaling off. In the presence of ABA, PYR/PYL/RCAR receptors bind the hormone, allowing them to physically associate with PP2Cs and eliminate the inhibitory effect of the phosphatases on SnRK2s, thereby switching ABA signaling on. Thus, SnRK2s are central components that positively regulate ABA signaling, and the regulation of SnRK2 kinase activity is crucial for turning ABA signaling on/off and for subsequent plant responses to abiotic stress. Thus, a fundamental question is how the ABA signaling pathway is controlled via the regulation of SnRK2s activity.

The activity of a protein kinase is mainly regulated at the transcriptional and protein levels [6, 7]. At the protein level, the activity of a kinase is determined by protein modification and its level of expression. Among the ten SnRK2s in *Arabidopsis*, SnRK2.2 (SnRK2D), SnRK2.3 (SnRK2I), and SnRK2.6 (SnRK2E or OST1) are highly similar in terms of their protein sequences and redundantly regulate many ABA-mediated processes, including seed dormancy, seed germination, seedling growth, and drought tolerance [6–8]. The transcriptional regulation of these genes and their contribution to SnRK2.2/2.3/2.6 activity are poorly understood. Regarding regulation at the protein level, current research has focused exclusively on the phosphorylation of SnRK2.2/2.3/2.6 [9]. SnRK2.2/2.3/2.6 are phosphorylated by autophosphorylation and/or other kinases, such as brassinosteroid (BR)-insensitive 2 (BIN2), casein kinase 2 (CK2), and ABA and abiotic stress-responsive Raf-like kinase (ARK) that have been proved to regulate SnRK2s activity and stability [7, 9–14]. In addition to protein modification, protein degradation plays a key role in regulating the mediators of many biological processes that allow plants to respond appropriately to cellular signals and environmental cues [15–17]. Unfortunately, it remains unclear how these SnRK2s are destabilized or targeted for degradation.

The ubiquitin-proteasome system is a major mechanism targeting specific subsets of proteins for degradation and controlling protein homeostasis in plant cells [15, 18, 19]. The ubiquitin 26S proteasome has four components: E1, E2, E3, and the 26S proteasome. In *Arabidopsis*, there are more than 1400 E3 ligases that are divided into two groups: single and multiple subunit ligases. Really Interesting New Gene (RING) type E3 ligases belong to the single subunit group, while cullin-based E3 ligases, e.g. the Skp1-CULLIN1-F-box (SCF) E3 ligase complex, CUL3-Broad-complex/Tramtrack/Bric-a-Brac, and CUL4-DNA damage-binding protein 1 belong to the multiple subunit group [16]. It has been demonstrated that ubiquitin/proteasome-dependent protein degradation plays an important role in the regulation of ABA signaling. For example, CUL4-based ligases and KEEP ON GOING (a RING E3 ligase) target ABI5 for degradation [20, 21], while the single subunit E3 ligase ABI3-interacting protein 2 controls the ABI3 protein level [22]. Meanwhile, the U-box E3 ligases PUB12 and PUB13 interact with PP2Cs to regulate the ABI1 protein level following ABA receptor binding [23]. Recent studies have also demonstrated that ubiquitin-based protein degradation is involved in ABA receptor degradation. This includes the DDA1-mediated degradation of PYL8, PYL4, and PYL9 [24, 25], and the SCF type E3 ligase RCAR3 INTERACTING F-BOX PROTEIN 1-mediated degradation of RCAR3, an ABA receptor [26]. A recent proteomic analysis found that SnRK1.1, SnRK2.4 and SnRK2.6 were the targets of ubiquitination, suggesting that these proteins may be degraded by ubiquitin proteasome system [27]. However, there is currently no report on the detailed mechanism for the degradation of SnRK2s.

SCF ubiquitin ligases are recognized for their strong substrate specificity, and F-box proteins help mediate substrate targeting [28]. In *Arabidopsis*, phloem protein 2-B11 (AtPP2-B11) is an F-box protein that acts as the substrate receptor for SCF type E3 ligases and which regulates LEA protein stability in drought-exposed plants [29]. AtPP2-B11 also affects the annexin1 protein level to positively regulate the response of plants to salt stress [30]. In this study, we show that AtPP2-B11 specifically targets SnRK2.3 for degradation. *AtPP2-B11* was induced by ABA, and *AtPP2-B11* knockdown plants were hypersensitive to ABA during seed germination and cotyledon greening. Intriguingly, AtPP2-B11 interacted directly with SnRK2.3 and accelerated SnRK2.3 degradation *in vivo* and *in vitro*. To our knowledge, our results reveal for the first time that SnRK2.2, SnRK2.3, and SnRK2.6 are regulated separately, and that SCF^AtPP2-B11^ controls plant responses to ABA by targeting SnRK2.3 for degradation. Therefore, our findings uncover a novel layer of the regulatory mechanism that dynamically modulates ABA signaling.

## Results

### SnRK2s are degraded by the 26S proteasome

**Fig 1:**
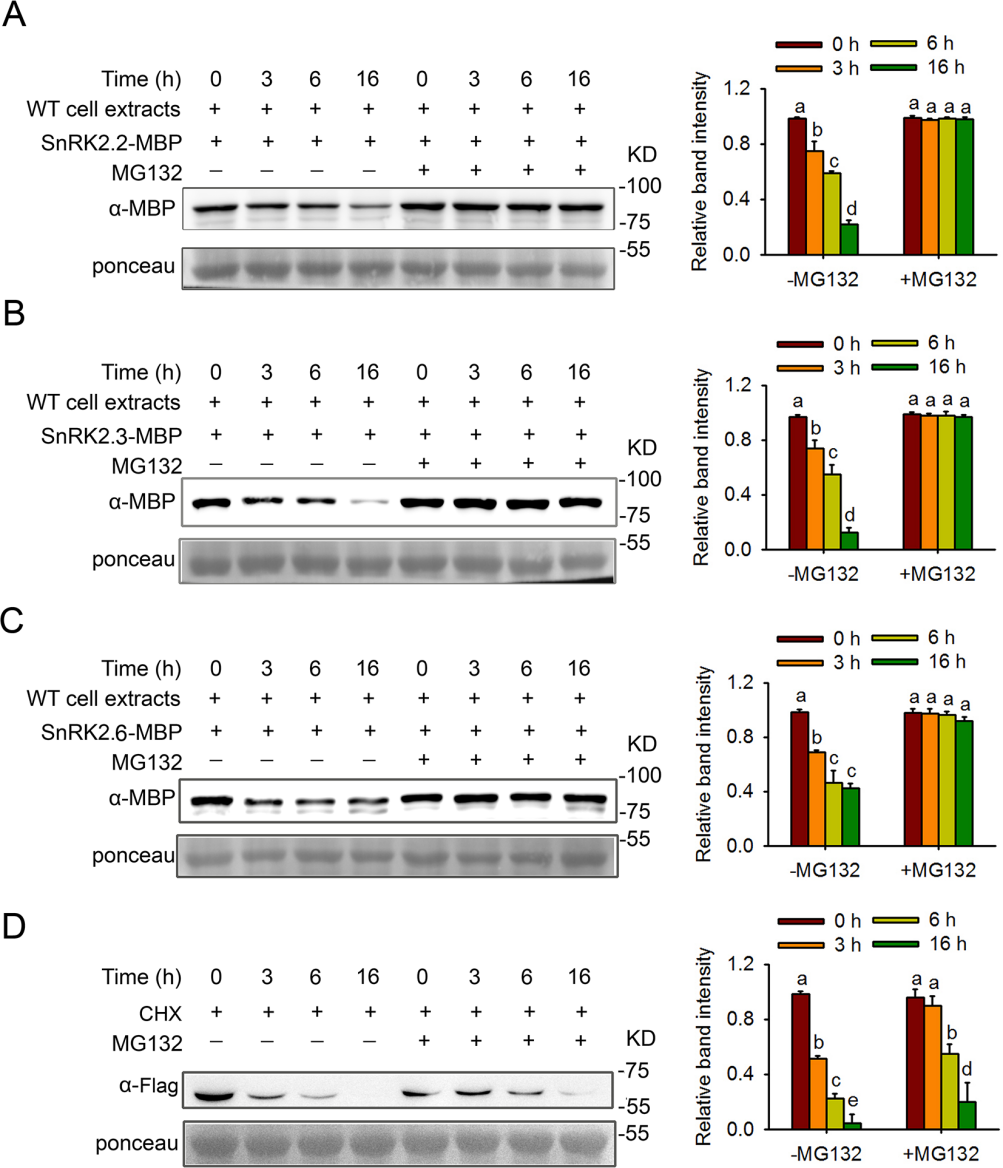
Cell-free assays for the degradation of SnRK2s in wild-type plants treated with or without MG132. Purified SnRK2.2-MBP (A), SnRK2.3-MBP (B), or SnRK2.6-MBP (C) was incubated with proteins extracted from wild-type plants for the indicated time period with or without MG132 treatment. Protein levels were checked using monoclonal anti-MBP antibody. Ponceau staining was used as loading control. Relative amounts of proteins were determined by ImageJ and normalized to loadings determined by Ponceau staining and expressed relative to the value at 0 hr time. Different letters indicate a significant difference (Student-Newman–Kuels [SNK] test, P < 0.05). Quantitative analysis of the band intensity was on the right side of the figure. Error bars are means ± s.e.m. (n ≥ 3 independent experiments). (D). *In vivo* degradation of SnRK2.3. *35S*::*SnRK2*.*3-3Flag* (*SnRK2*.*3-OE-8*) seedlings were treated with 50 μM CHX (protein biosynthesis inhibitor) or 50 μM CHX and 50 μM MG132 separately for different times before protein was isolated for western blot with anti-Flag antibody. Ponceau staining was used as loading control. Proteins were detected as in (A-C). Different letters indicate a significant difference (Student-Newman–Kuels [SNK] test, P < 0.05). Quantitative analysis of the band intensity was on the right side of the figure. Error bars are means ± s.e.m. (n ≥ 3 independent experiments).

To investigate the degradation characteristics of SnRK2s, we examined whether SnRK2 proteins undergo degradation and whether they are degraded via the ubiquitin-proteasome pathway using a cell-free system. We expressed and purified SnRK2.2, SnRK2.3, and SnRK2.6 tagged with maltose-binding protein (MBP) in BL21 *Escherichia coli* cells, and then incubated those SnRK2s with total protein extracts prepared from 7-day-old *Arabidopsis* seedlings grown on Murashige and Skoog (MS) medium. When incubated with the total protein extracts without MG132 (a 26S proteasome inhibitor), the levels of all of the SnRK2s were low at 3 h after incubation, and the protein levels decreased gradually over time (Fig 1A–1C). By contrast, the SnRK2s incubated with proteins containning MG132 were stable, and the protein levels of the kinases were substantially elevated (Fig 1A–1C). These results indicate that SnRK2 proteins are actively degraded by the 26S proteasome.

To further confirm the degradation of SnRK2s, we took SnRK2.3 as an example to study the degradation *in vivo*. We overexpressed SnRK2.3 in wild type tagged with Flag and obtained two stable transgenic lines *SnRK2*.*3-OE-1* and *SnRK2*.*3-OE-8* (S1 Fig). We used *SnRK2*.*3-OE-8* seedlings to detect SnRK2.3 protein level with CHX or CHX and MG132 treatment at indicated time. We found that the degradation of SnRK2.3 is much slower when treated with CHX and MG132 compared that with CHX (Fig 1D), suggesting that SnRK2.3 is indeed degraded by the 26S proteasome.

### SnRK2.2 and SnRK2.3, but not SnRK2.6, interact with AtPP2-B11

To identify E3 ubiquitin ligases affecting the degradation/stability of SnRK2 proteins, we performed a yeast two-hybrid screening using SnRK2.6 as bait. Intriguingly, we identified AtPP2-B11, an F-box family protein, as the most likely candidate that may be involved in the regulation of SnRK2.6 protein stability. Previous yeast two-hybrid results have shown that AtPP2-B11 interacts with SKP1A and SKP1B, which are core components of SCF family E3 ubiquitin ligases [31]. This result prompted us to investigate in greater detail whether AtPP2-B11 regulates SnRK2.6 stability.

**Fig 2:**
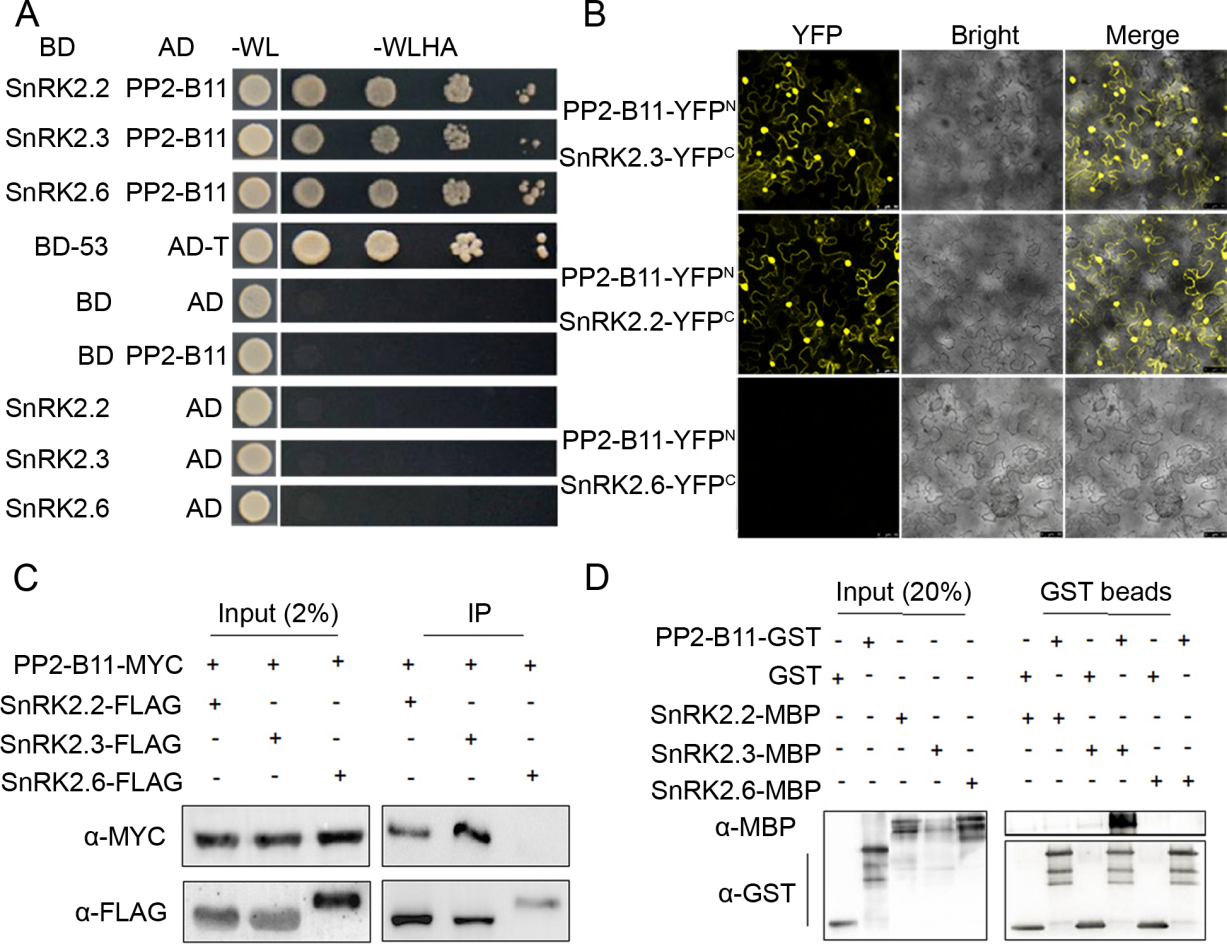
Assay for the interaction of AtPP2-B11 with SnRK2.2, SnRK2.3, or SnRK2.6. (A). The F-box protein AtPP2-B11 was found to interact with the protein kinases SnRK2.2, SnRK2.3, and SnRK2.6 by yeast two-hybrid growth assays performed on synthetic dropout medium lacking tryptophan and leucine (-WL) and synthetic dropout medium lacking tryptophan, leucine, histidine and adenine (-WLHA). Saturated cultures were spotted onto SD-Trp-Leu-His-Ade medium at different dilutions (OD_600_ = 1, 10^−1^, 10^−2^, and 10^−3^). The vectors AD-T and BD-53 were used as positive controls; the empty vectors pGADT7 and pGBKT7 were used as negative controls. (B). The interactions between AtPP2-B11-YFP^N^ and SnRK2.2/ SnRK2.3/ SnRK2.6-YFP^C^ in *N*. *benthamiana* were analyzed using BiFC assays. The YFP signal (left), brightfield images (middle), and merged images (right) are shown. (C). *In vivo* interaction assays for SnRK2.2/SnRK2.3/SnRK2.6-FLAG and AtPP2-B11-Myc by Co-IP in stably transformed *Arabidopsis* plants. Proteins were extracted from 7-day-old seedlings and incubated with agarose-conjugated monoclonal anti-Flag antibodies; AtPP2-B11-Myc was detected using monoclonal anti-myc antibodies. Input, 2% of the protein extract used in the Co-IP assay without IP. (D). *In vitro* pull-down assay for AtPP2-B11 with SnRK2.2, SnRK2.3, and SnRK2.6. SnRK2.2-MBP/SnRK2.3-MBP/SnRK2.6-MBP pulled down with AtPP2-B11-GST were detected using anti-MBP antibodies. Input, 20% of the purified GST- and MBP-tagged proteins used in the pull-down assays.

To this end, we first validated the interaction between SnRK2.6 and AtPP2-B11 using *in vitro* and *in vivo* assays. Because SnRK2.6 shares high levels of protein similarity with SnRK2.2 and SnRK2.3 [31], we also included these two proteins in the assays to determine whether AtPP2-B11 specifically regulates SnRK2.6 stability. Strong interactions were detected between AtPP2-B11 and all of the SnRK2s in yeast two-hybrid assay (Fig 2A). We next performed a bimolecular fluorescence complementation (BiFC) assay using a transient expression system in *Nicotiana benthamiana* leaf cells. Unexpectedly, no interaction was observed between AtPP2-B11 and SnRK2.6, but a strong YFP signal was detected in both the nucleus and cytoplasm between AtPP2-B11 and SnRK2.2 and SnRK2.3, and no YFP signal was detected in the plant cells expressing the control constructs (Fig 2B and S2 Fig). To verify this result, we performed a co-immunoprecipitation (Co-IP) assay. No AtPP2-B11-SnRK2.6 protein interaction was detected (Fig 2C); however, AtPP2-B11-SnRK2.2 and AtPP2-B11-SnRK2.3 interactions were detected in the Co-IP assay (Fig 2C). To investigate the direct interaction between AtPP2-B11 and SnRK2.2/SnRK2.3, we performed a pull-down assay. Only SnRK2.3 interacted with AtPP2-B11 (Fig 2D). These data demonstrate a strong direct interaction between AtPP2-B11 and SnRK2.3, but no direct interaction with SnRK2.2 or SnRK2.6.

AtPP2-B11, which is annotated as an SCF E3 ligase complex component, may mediate the ubiquitination and degradation of specific target proteins. AtPP2-B11 consists of 257 amino acids and contains a conserved F-box domain at its N-terminus and a phloem protein (PP) domain at its C-terminus (S3 Fig). Previously, it was shown that AtPP2-B11 is a cytoplasmic protein [29]. The fact that AtPP2-B11 interacts with SnRK2.2 and SnRK2.3 in both the cytoplasm and nucleus suggests that it is widely distributed in plant cells. To test this, we expressed *GFP*:*AtPP2-B11* under the control of the CaMV35S promoter in *N*. *benthamiana* leaf cells and *Arabidopsis*, respectively. Strong GFP fluorescence was observed in both the cytoplasm and nucleus in plant cells (S4 Fig), confirming that AtPP2-B11 is a widely distributed protein.

Previous comprehensive analyses of protein-protein interactions have revealed an interaction between AtPP2-B11 and ASK1 or ASK2 [32]. To confirm that AtPP2-B11 functions in the SCF E3 ligase complex, we tested for a physical interaction between AtPP2-B11 and ASK1 or ASK2. Yeast two-hybrid assays revealed a strong interaction between AtPP2-B11 and ASK1 or ASK2 (S5A Fig). We then performed a BiFC assay to assess the interactions of AtPP2-B11-ASK1 and AtPP2-B11-ASK2 in plant cells. AtPP2-B11 was fused to the N-terminal end of YFP while ASK1 and ASK2 were fused to the C-terminal end of YFP. When the two constructs were coexpressed in *N*. *benthamiana* leaf cells, there was a strong reconstituted YFP signal in both the nucleus and cytoplasm; by contrast, no YFP signal was detected in the plant cells expressing the control constructs (S5B Fig and S6 Fig). These results indicate that AtPP2-B11 is indeed a component of the SCF E3 ligase complex and that it may mediate the degradation of target proteins in both the nucleus and cytoplasm.

### AtPP2-B11 affects the stability of SnRK2.3, but not SnRK2.2 or SnRK2.6

AtPP2-B11 functions as a receptor for proteins that are destined for degradation. Since AtPP2-B11 interacts with SnRK2.2 and SnRK2.3 in plant cells, we questioned whether AtPP2-B11 mediates the degradation of these SnRK2s. To answer this question, we analyzed the effects of AtPP2-B11 expression on the stability of SnRK2s. We first generated *35S*::*AtPP2-B11-Myc* overexpression (*AtPP2-B11OE*) lines and *AtPP2-B11* knockdown (*amiR AtPP2-B11*) lines *amiR7* and *amiR15* (S7A and S7B Fig). Those *AtPP2-B11OE* and *amiR AtPP2-B11-7/15* plants that displayed increased or reduced levels of *AtPP2-B11* expression were selected for further analysis. Notably, these transgenic plants were comparable to wild-type Col-0 in terms of plant growth and development (S7C and S7D Fig).

**Fig 3:**
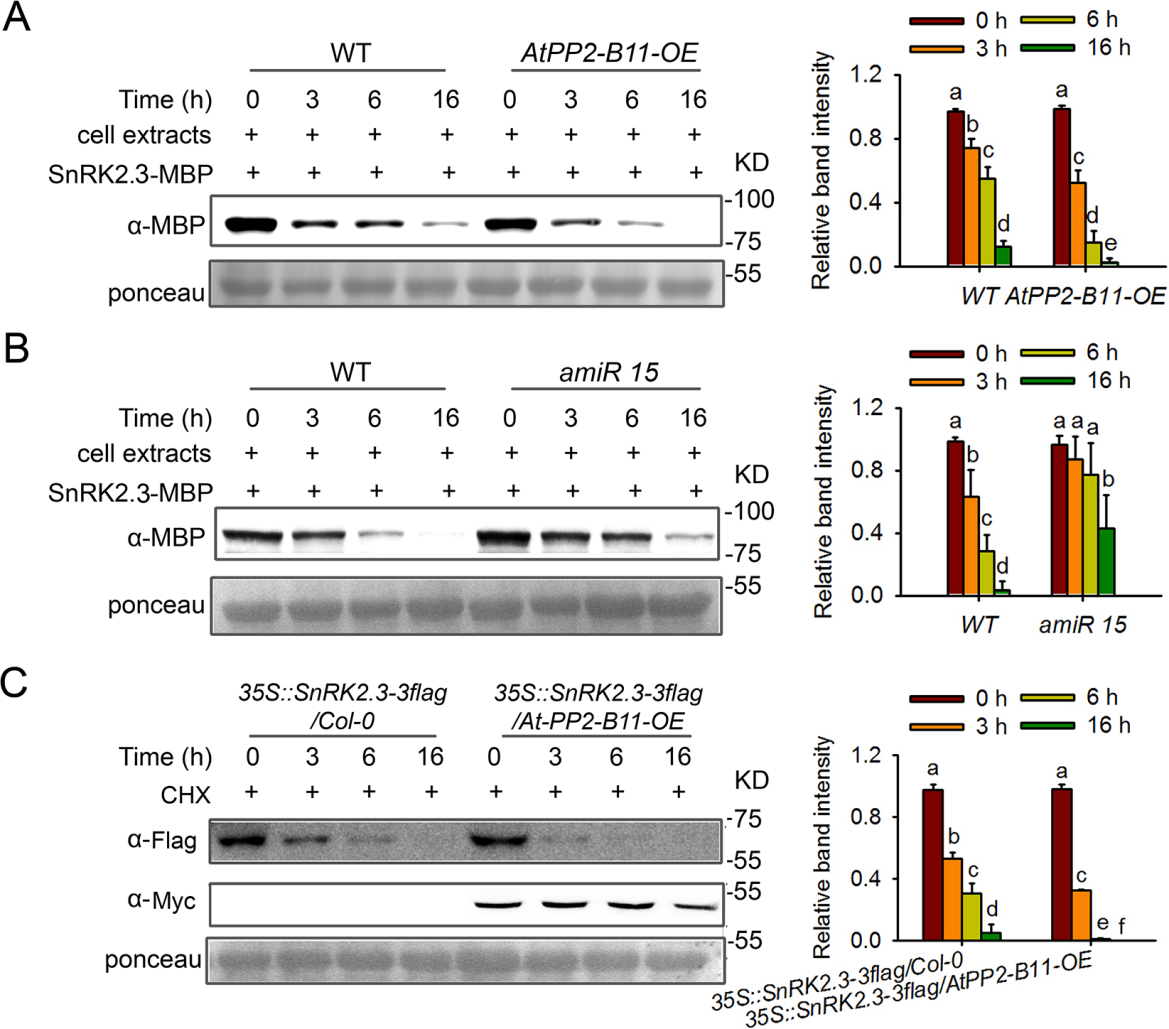
AtPP2-B11 affects the stability of SnRK2.3, but not SnRK2.2 or SnRK2.6. (A). Assays for the degradation of MBP-tagged SnRK2.3 were performed using wild-type plants and AtPP2-B11-overexpressing (OE) transgenic lines. Ponceau staining was used as loading control. Relative amounts of proteins were determined by ImageJ and normalized to loadings determined by Ponceau staining and expressed relative to the value at 0 hr time. Different letters indicate a significant difference (Student-Newman–Kuels [SNK] test, P < 0.05). Quantitative analysis of the band intensity was on the right side of the figure. Error bars are means ± s.e.m. (n ≥ 3 independent experiments). (B). Assays for the degradation of MBP-tagged SnRK2.3 were performed using wild-type plants and the *amiR-AtPP2-B11* (*amiR15*) transgenic line. The plants were pre-treated by 50 μM ABA for 5 h. Ponceau staining was used as loading control. Proteins were detected as in (A). Different letters indicate a significant difference (Student-Newman–Kuels [SNK] test, P < 0.05). Quantitative analysis of the band intensity was on the right side of the figure. Error bars are means ± s.e.m. (n ≥ 3 independent experiments). (C). The degradation assay for *35S*::*SnRK2*.*3-3flag* in wild type or *AtPP2-B11* overexpression background. Proteins were extracted from 7-day-old transgenic seedlings with 50 uM CHX (protein biosynthesis inhibitor) treatment for indicated times. The SnRK2.3 protein level was checked at the indicated time point by western blotting using anti-Flag antibody. AtPP2-B11-Myc protein level was detected using monoclonal anti-myc antibody. Ponceau staining was used as loading control. Proteins were detected as in (A). Different letters indicate a significant difference (Student-Newman–Kuels [SNK] test, P < 0.05). Quantitative analysis of the band intensity was on the right side of the figure. Error bars are means ± s.e.m. (n ≥ 3 independent experiments).

Next, we performed a cell-free degradation assay for SnRK2s. Because AtPP2-B11 interacted most strongly with SnRK2.3 in our protein-protein interaction assays, we assessed SnRK2 degradation using SnRK2.3 as an example. SnRK2.3-MBP was expressed and purified in *E*. *coli* and then incubated with total proteins extracted from wild-type or *AtPP2-B11OE* plants. As shown in Fig 3A, the level of SnRK2.3-MBP was markedly decreased at 3, 6, and 16 h after incubation with proteins from *AtPP2-B11OE* plants compared to proteins from wild-type plants (Fig 3A). We also tested the degradation of SnRK2.3 protein incubated with proteins from *amiR15* plants. As *AtPP2-B11* expression was increased significanty with ABA treatment (S7B Fig), we firstly treated the wild type and *amiR15* plants with 50 μM ABA for 5 h and then extracted the total proteins for degradation assay. In sharp contrast, when SnRK2.3-MBP was incubated with proteins extracted from *AtPP2-B11* mutant *amiR15* plants, the SnRK2.3-MBP level was much higher than that following incubation with wild-type proteins (Fig 3B). To confirm this result, we generated transgenic plants of *35S*::*SnRK2*.*3-Flag* (*SnRK2*.*3-OE-8*) in wild type or *AtPP2-B11-Myc* overexpression (*AtPP2-B11OE*) background (S8 Fig). The overexpression of *AtPP2-B11* expression resulted in a decrease in the SnRK2.3 protein level (Fig 3C). To confirm the result futher, we ordered a T-DNA insertion line GK-162G12 (named *atpp2-b11*) from the Nottingham *Arabidopsis* Stock Center. The T-DNA is inserted in the first exon of *AtPP2-B11* and the homozygous mutants were identified by PCR using *AtPP2-B11* gene-specific and T-DNA border primers. RT-PCR analysis verified that there is no full length transcript of *AtPP2-B11* in *atpp2-b11* mutant (S9 Fig). We then performed a cell-free degradation assay for SnRK2.3 using wild type and knock-out mutant *atpp2-b11*. SnRK2.3-MBP was incubated with proteins extracted from wild type and knock-out mutant pre-treated by 50 μM ABA for 5 h. We found SnRK2.3-MBP protein level incubated with proteins extracted from *atpp2-b11* knock-out mutant was much higher than that incubated with wild-type proteins (S10 Fig). These results demonstrate that AtPP2-B11 promotes SnRK2.3 degradation.

To further validate whether AtPP2-B11 promotes the degradation of SnRK2.2 and SnRK2.6, we tested the influence of AtPP2-B11 on levels of SnRK2.2/2.6 proteins using cell-free degradation assay. The recombinant SnRK2.2-MBP or SnRK2.6-MBP were incubated with proteins extracted from the wild type, *AtPP2-B11 OE* or *amiR15* plants. The result showed that the levels of SnRK2.2-MBP or SnRK2.6-MBP were comparable when incubated with the proteins from wild type, *AtPP2-B11 OE* or *amiR15* plants treatment (S11 Fig), suggesting that AtPP2-B11 does not affect the degradation of SnRK2.2/2.6 in the cell free system. Thus, AtPP2-B11 specifically promotes the degradation of SnRK2.3 in *Arabidopsis* plants.

### ABA promotes the protein degradation of SnRK2.3

**Fig 4:**
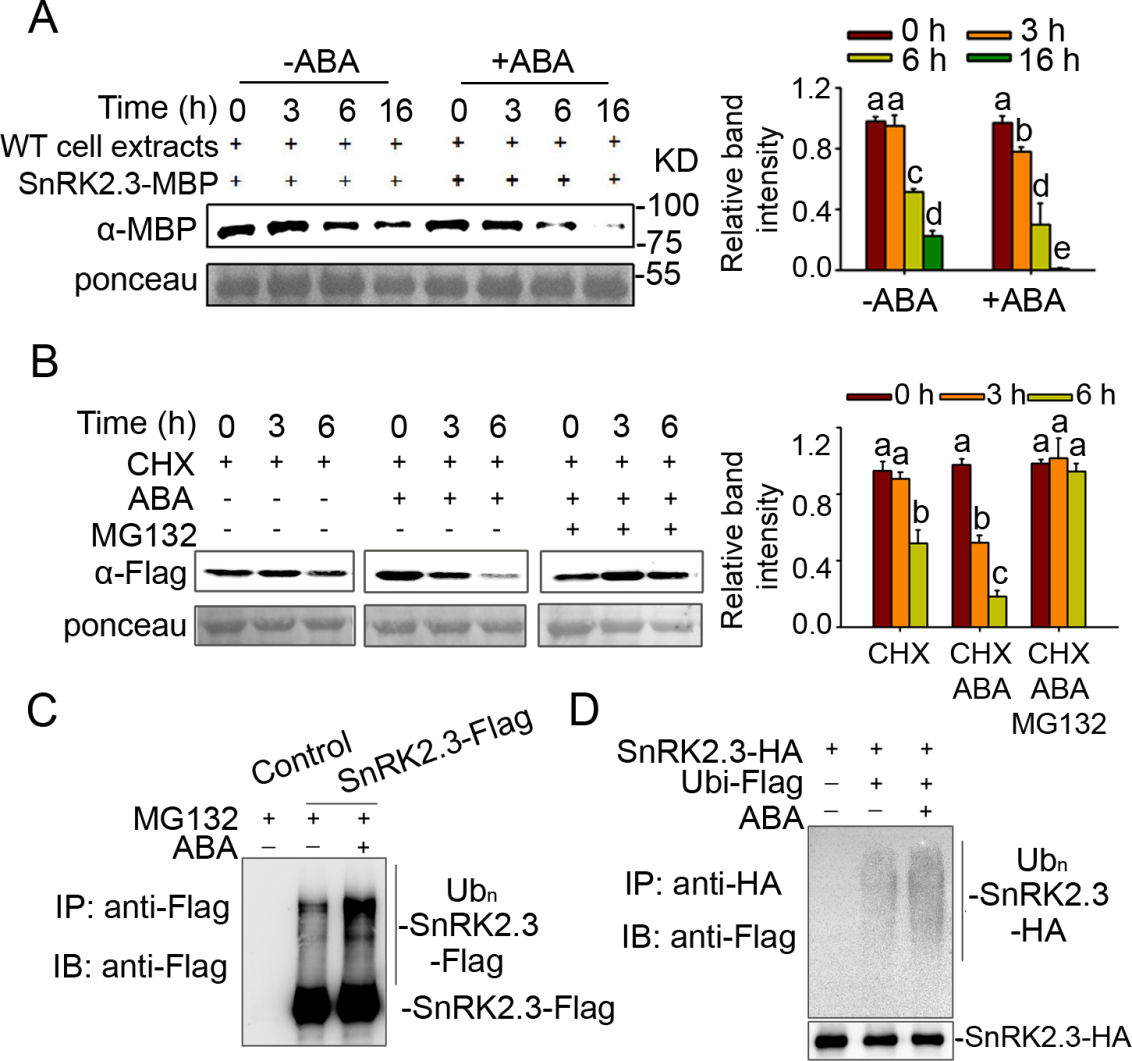
ABA promotes the degradation of SnRK2.3. (A). Assays for SnRK2.3 degradation *in vitro*. SnRK2.3-MBP was incubated with proteins extracted from wild type plants treated with or without 50 μM ABA for 5 h. SnRK2.3 protein was detected with anti-MBP antibody. Ponceau staining was used as loading control. Relative amounts of proteins were determined by ImageJ and normalized to loadings determined by Ponceau staining and expressed relative to the value at 0 hr time. Different letters indicate a significant difference (Student-Newman–Kuels [SNK] test, P < 0.05). Quantitative analysis of the band intensity was on the right side of the figure. Error bars are means ± s.e.m. (n ≥ 3 independent experiments). (B). The ABA-induced SnRK2.3 degradation is dependent on the 26S proteasome pathway. Seven-day-old transgenic seedlings were treated for indicated time points with 50 μM CHX, 50 μM CHX with 50 μM ABA, or 50 μM CHX together with 50 μM ABA and 50 μM MG132, respectively. The levels of SnRK2.3-Flag at each time points were detected by anti-Flag antibody. Ponceau staining was used as loading control. Relative amounts of proteins were determined by ImageJ and normalized to loadings determined by Ponceau staining and expressed relative to the value at 0 h time. Different letters indicate a significant difference (Student-Newman–Kuels [SNK] test, P < 0.05). Quantitative analysis of the band intensity was on the right side of the figure. Error bars are means ± s.e.m. (n = 3 independent experiments). (C). The ABA-induced degradation of SnRK2.3 is mediated by ubiquitin-dependent proteasomal degradation. Seven-day-old *SnRK2*.*3-Flag* transgenic seedlings and the wild type seedlings were treated with 50 μM CHX and with 50 μM ABA or not for 9 h. SnRK2.3-Flag protein was isolated using Flag beads (ANTI-FLAG M2 Affinity Gel; Sigma-Aldrich). Flag antibody was used to detect SnRK2.3 and the ubiquitinated level of SnRK2.3. IP: immunoprecipitation; IB: immunoblot. (D). Ubiquitination of SnRK2.3-HA treated with ABA or not in protoplasts. Arabidopsis wild type protoplasts were transformed with SnRK2.3-HA or SnRK2.3-HA together with Ubiquitin-Flag (Ubi-Flag), incubated for 8 h of protein synthesis, then treated with 50 μM MG132 for 1 h, and then finally treated with or without 20 μM ABA for another 2 h. Proteins were isolated for immunoprecipitation with HA antibody for 2 h at 4°C, and then incubated with protein A beads for another 2 h at 4°C, and followed by immunoblotting with anti-Flag and anti-HA antibodies to detect the ubiquitinated levels of SnRK2.3 and SnRK2.3 protein levels, respectively.

ABA activates the kinase activity of SnRK2.3, at the same time, ABA also induces the gene expression of *AtPP2-B11*. We wondered whether ABA affects the stability of SnRK2.3. To answer this question, we performed cell-free degradation assay using 7-day-old wild type seedlings treated with or without 50 μM ABA for 5 h. We found the SnRK2.3 protein level incubated with proteins extracted from ABA-treated seedlings was much lower than that incubated with proteins from the seedlings without ABA treatment (Fig 4A).

To confirm whether the effect of ABA on the degradation of SnRK2.3 is dependent on the 26S proteasome pathway, 7-day-old *SnRK2*.*3-OE* transgenic seedlings were treated for indicated time with 50 μM CHX, 50 μM CHX and 50 μM ABA, or 50 μM CHX and 50 μM ABA together with 50 μM MG132, respectively. SnRK2.3 protein levels at different time points were detected with Flag antibody. As shown in Fig 4B, degradation of the SnRK2.3 protein in CHX-treated seedlings was much slower than that of the plants treated with both CHX and ABA. However, when the seedlings simultaneously treated with CHX, ABA and MG132, the SnRK2.3 protein degradation was completely inhibited (Fig 4B). This result suggests that ABA promotes SnRK2.3 degradation and the ABA-induced SnRK2.3 degradation is dependent on the 26S proteasome pathway.

To confirm that ABA-induced degradation of SnRK2.3 is mediated by ubiquitin-dependent proteasomal degradation, 7-day-old *SnRK2*.*3-Flag* transgenic seedlings were treated with 50 μM MG132 supplementing with or without 50 μM ABA for 9 h. SnRK2.3-Flag protein was then immunoprecipitated using Anti-Flag M2 Affinity Gel for detection of SnRK2.3-Flag ubiquitination level. The result showed that the ubiquitinated level of SnRK2.3 in the seedlings treated with MG132 was much lower than that treated with MG132 and ABA (Fig 4C). To further confirm this result, we transformed wild type leaf protoplasts with plasmids expressing SnRK2.3-HA, or SnRK2.3-HA and Ubiquitin-Flag. After 8 h incubation, the protoplasts were treated first with MG132 for 1 h, then with or without ABA for another 2 h (Fig 4D). As shown in Fig 4D, the ubiquitinated level of SnRK2.3 was fairly low without ABA; by contrast, the level of ubiquitinated SnRK2.3 was markedly increased under ABA treatment (Fig 4D). These results demonstrate that ABA promotes the ubiquitin modification and the degradation of SnRK2.3.

### *AtPP2-B11* is expressed and induced by ABA in multiple tissues/organs

**Fig 5:**
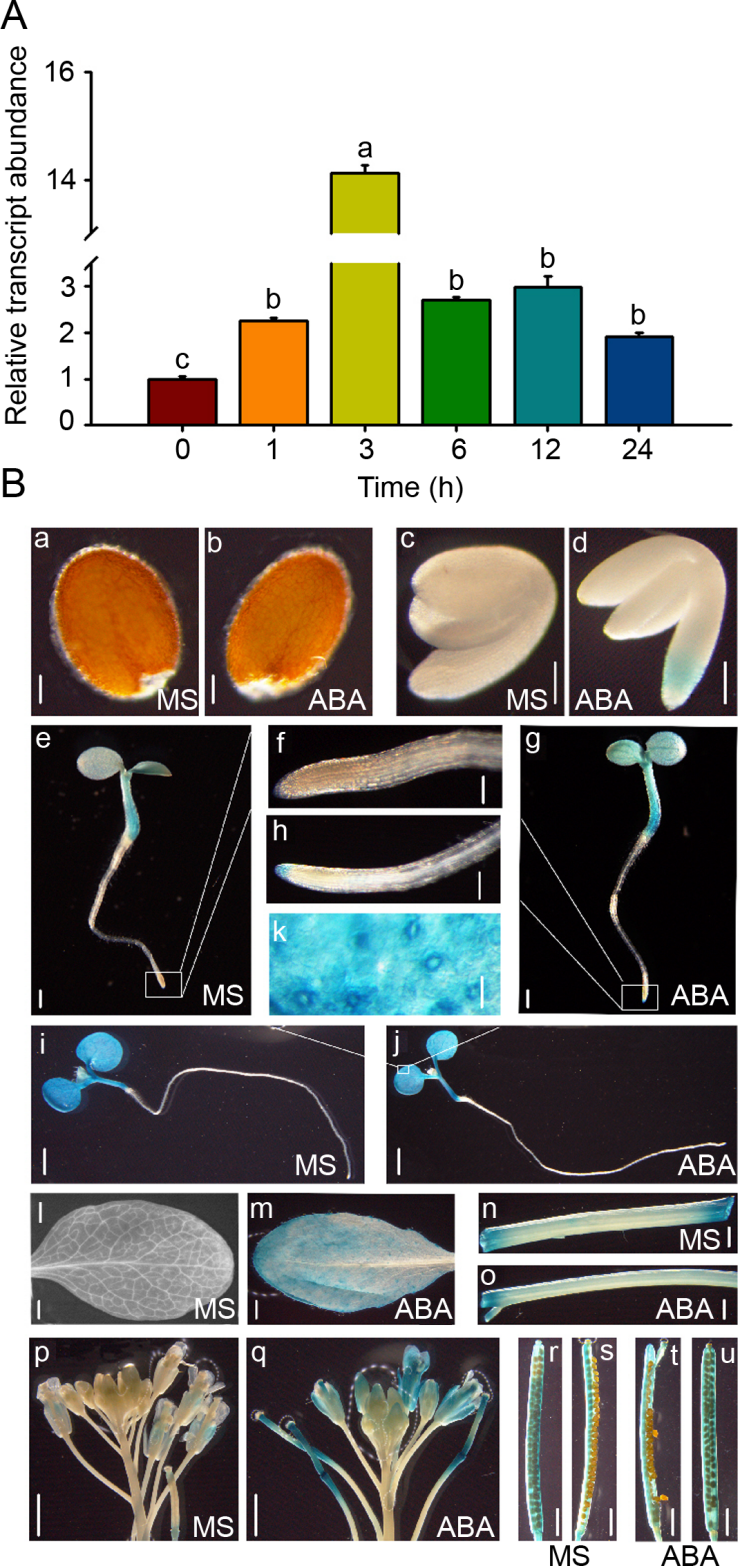
Expression pattern of *AtPP2- B11*. (A). *AtPP2-B11* expression assay in response to ABA by quantitative real-time PCR. Total RNA was extracted from 7-day-old wild-type seedlings treated with 50 μM ABA at the indicated time points. *ACTIN2* was used as an internal control. The values are means±standard error. Different letters indicat a significant difference (Student-Newman–Kuels [SNK] test, P < 0.05). Three independent biological repeats were performed. (B). GUS staining of the *pAtPP2-B11*::*GUS* transgenic lines with or without 50 μM ABA treatment for 3 h. (a and b) Imbibition seeds. (c and d) One-day-old imbibed seeds. (e and g) Three-day-old seedlings. (f and h) Amplified view of root tip of (e) and (g). (i and j) Five-day-old seedlings. (k) Amplified view of a leaf. (l and m) Rosette leaves of 3-week-old seedlings. (n and o) Stems of 3-week-old seedlings. (p and q) Inflorescences. (r and u) Siliques. (s and t) Immature seeds from siliques. Scale bars = 0.1 mm (a–d), 0.05 mm (f, h, and k), 0.3 mm (l–o), and 1 mm (e, g, i, j, and p–u).

The fact that AtPP2-B11 specifically promoted SnRK2.3 protein degradation prompted us to investigate whether *AtPP2-B11* is responsive to ABA and modulates plant responses to ABA. To this end, we analyzed the expression pattern of *AtPP2-B11* in response to ABA in 7-day-old seedlings germinated on MS medium. Quantitative real-time PCR showed that *AtPP2-B11* was induced significantly by ABA and reached its highest level at 3 h after treatment (Fig 5A), consistent with the microarray data from a publicly available source (TAIR; S12 Fig). To understand transcription characteristics of *AtPP2-B11*, we analyzed the promoter of *AtPP2-B11*, and found that there are several ABRE (CACGTG) *cis*-elements in its promoter (S13 Fig), consistent with our result that *AtPP2-B11* expression was induced by ABA (Fig 5A). The *AtPP2-B11* promoter also contains MBS (TAACTG), LTR (CCGAAA), TCA (CAGAAAAGGA) and GARE (AAACAGA) *cis-*elements, indicating *AtPP2-B11* may also respond to drought, low tempreture, salicylic acid and gibberellin (S13 Fig). Next, we generated transgenic plants expressing *β-glucuronidase* (GUS) under the control of the native promoter of *AtPP2-B11*. Histochemical staining showed that *AtPP2-B11* exhibited a tissue- and organ-specific expression pattern, but unexpectedly, expression of *AtPP2-B11* was hardly detectable in phloem. We found that *AtPP2-B11* was responsive to ABA in some tissues and organs. In the absence of ABA, *AtPP2-B11* was expressed at a higher level in germinating young seedlings, including cotyledons and hypocotyls, but not roots (Fig 5Be and 5Bf). Interestingly, the level of *AtPP2-B11* expression was highest in the shoots of germinating seedlings when the cotyledons were fully expanded (Fig 5Bi). Low levels of *AtPP2-B11* expression were observed in the rosette leaves of young plants and in the stem and siliques of mature plants at the reproductive stage (Fig 5Bl, 5Bn, 5Br and 5Bs). Further, *AtPP2-B11* was induced by ABA specifically in the embryonic roots of imbibed seeds and in the root tips of germinating young seedlings (Fig 5Bd, 5Bg and 5Bh); ABA induction of *AtPP2-B11* was also observed in rosette leaves and flowers (Fig 5Bm and 5Bq). The expression pattern of *AtPP2-B11* treated by ABA reminds the expression of *SnRK2*.*3*, which is expressed in stems, roots, flowers, siliques and root tips [6], suggesting that the two genes have similar express pattern in plants.

### AtPP2-B11 is a negative regulator of ABA signaling

**Fig 6:**
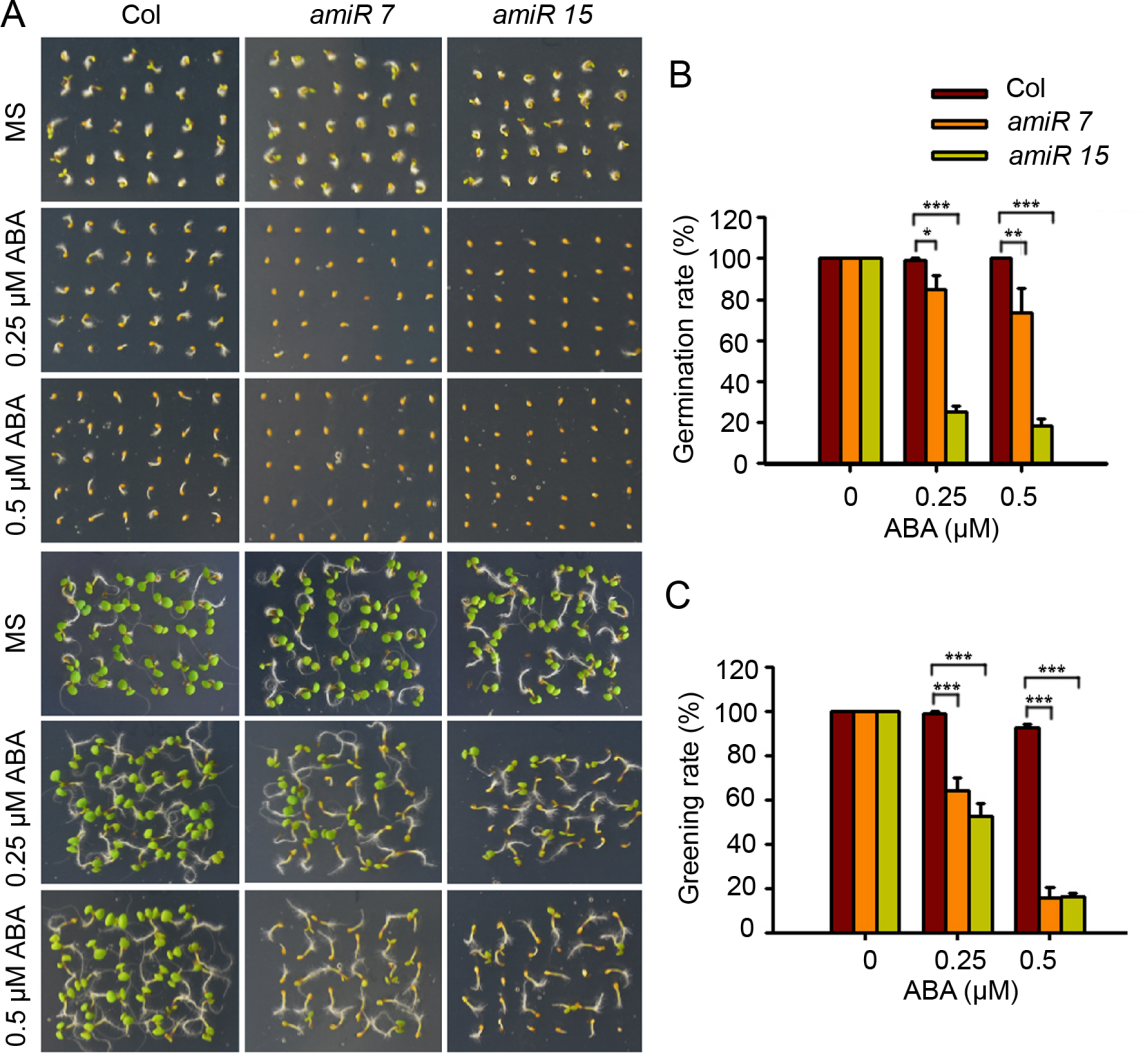
AtPP2-B11 is a negative regulator of ABA signaling. (A). Phenotypic analyses of wildtype (WT) and *AtPP2-B11* mutant plants treated with 0.25 or 0.5 μM ABA. The images were taken after 4 and 8 days, respectively. (B). The germination rates and (C). greening rates for WT, *amiR7*, and *amiR15* plants at 4 and 8 days after stratification. The data are given as means plus the standard deviation of three independent replicates. The student’s t-test was performed and the statically significant treatments were marked with ‘***’ (P<0.001), ‘**’ (P<0.01) and ‘*’ (P<0.05).

To investigate whether AtPP2-B11 is required for plant growth and ABA responses, we performed a phenotypic analysis of *AtPP2-B11* knockdown plants using artificial microRNA technology. Among the putative transgenic lines, we identified two lines (*amiR7* and *amiR15*) that showed significant reductions in *AtPP2-B11* expression following ABA treatment (S7B Fig). We then analyzed the phenotypes of these lines during early developmental stages in the absence or presence of ABA. Although *AtPP2-B11* was expressed in germinating seedlings, unexpectedly, there were no significant differences in seed germination and cotyledon greening between the *amiR7*/*amiR15* lines and wild type in the absence of ABA (Fig 6A). However, the *amiR7* and *amiR15* lines exhibited substantially increased sensitivity to ABA during early development. The germination and cotyledon greening rates of the *amiR7* and *amiR15* plants were much lower than that of wild type upon ABA treatment (Fig 6B and 6C). For example, at 0.5 μM ABA, 100% of the wild-type seeds had germinated at 4 days after treatment, whereas only 73% of the *amiR7* seeds and 18.3% of the *amiR15* seeds had germinated. Similarly, about 92.5% of wild-type germinating seedlings turned green, compared to only 15.8% of *amiR7* and 16.7% of *amiR15* germinating seedlings. Next, we analyzed the phenotype of knockout mutant *atpp2-b11* during early developmental stages in the absence or presence of ABA. We found that *atpp2-b11* mutant was hypersensitive to ABA in both germination and greening stages, which is consistent with the phenotypes of knockdown mutant *amiR7* and *amiR15* (S14 Fig). These results suggest that AtPP2-B11 serves as a negative regulator in ABA signaling.

### *AtPP2-B11* affects the expression of ABA-responsive genes

**Fig 7:**
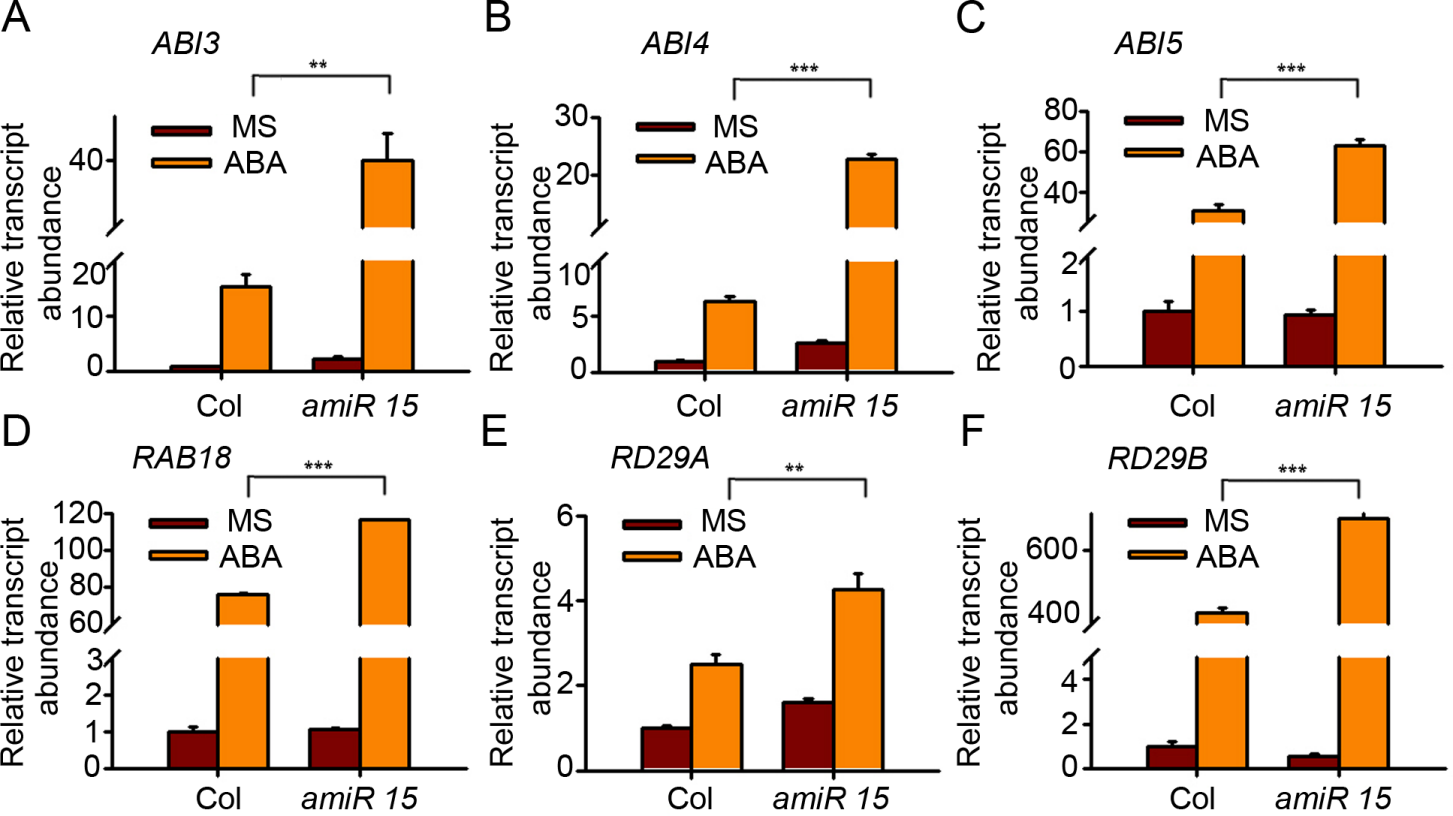
The transcript abundance of ABA-responsive genes in Col-0 and *amiR15*. The relative transcript abundance of (A) *ABI3*, (B) *ABI4*, (C) *ABI5*, (D) *RAB18*, (E) *RD29A*, and (F) *RD29B* in Col-0 and *amiR15* mutant plants were analyzed. Seedlings were grown on MS medium with or without 0.5 μM ABA for 10 days. Three independent experiments were performed with similar results, each with three replicates. *UBC5* was used as the internal control. The student’s t-test was performed and the statically significant treatments were marked with ‘***’ (P<0.001) and ‘**’ (P<0.01).

Given that AtPP2-B11 promotes the degradation of SnRK2.3, which positively regulates ABA signaling, we predicted that AtPP2-B11 must negatively regulate the expression of ABA-responsive genes downstream of SnRK2.3. To prove this, we analyzed the effects of AtPP2-B11 on the expression of *ABI3*, *ABI4*, *ABI5*, *RAB18*, *RD29A*, and *RD29B*. 10-day-old Col-0 and *amiR15* seedlings germinated on MS medium with or without 0.5 μM ABA were used for RNA extraction and gene expression analysis. In the wild-type, all the genes were induced by ABA, consistent with previous findings [33–35]. In the *amiR15* seedlings, *ABI3*, *ABI4*, *ABI5*, *RAB18*, *RD29A*, and *RD29B* were induced by ABA, but the transcript abundance of these genes were greatly elevated compared with those in wild-type plants treated with ABA (Fig 7). We also checked the ABA response genes in the knock-out mutant *atpp2-b11*, and the result also showed that the transcript abundance of all the ABA response genes we tested were much higher in *atpp2-b11* mutant than that of wild type in the treatment of ABA (S15 Fig). These results confirmed the negative role of AtPP2-B11 in ABA signaling.

### *AtPP2-B11* overexpression inhibits the ABA hypersensitive phenotypes of *SnRK2*.*3* overexpression lines

**Fig 8:**
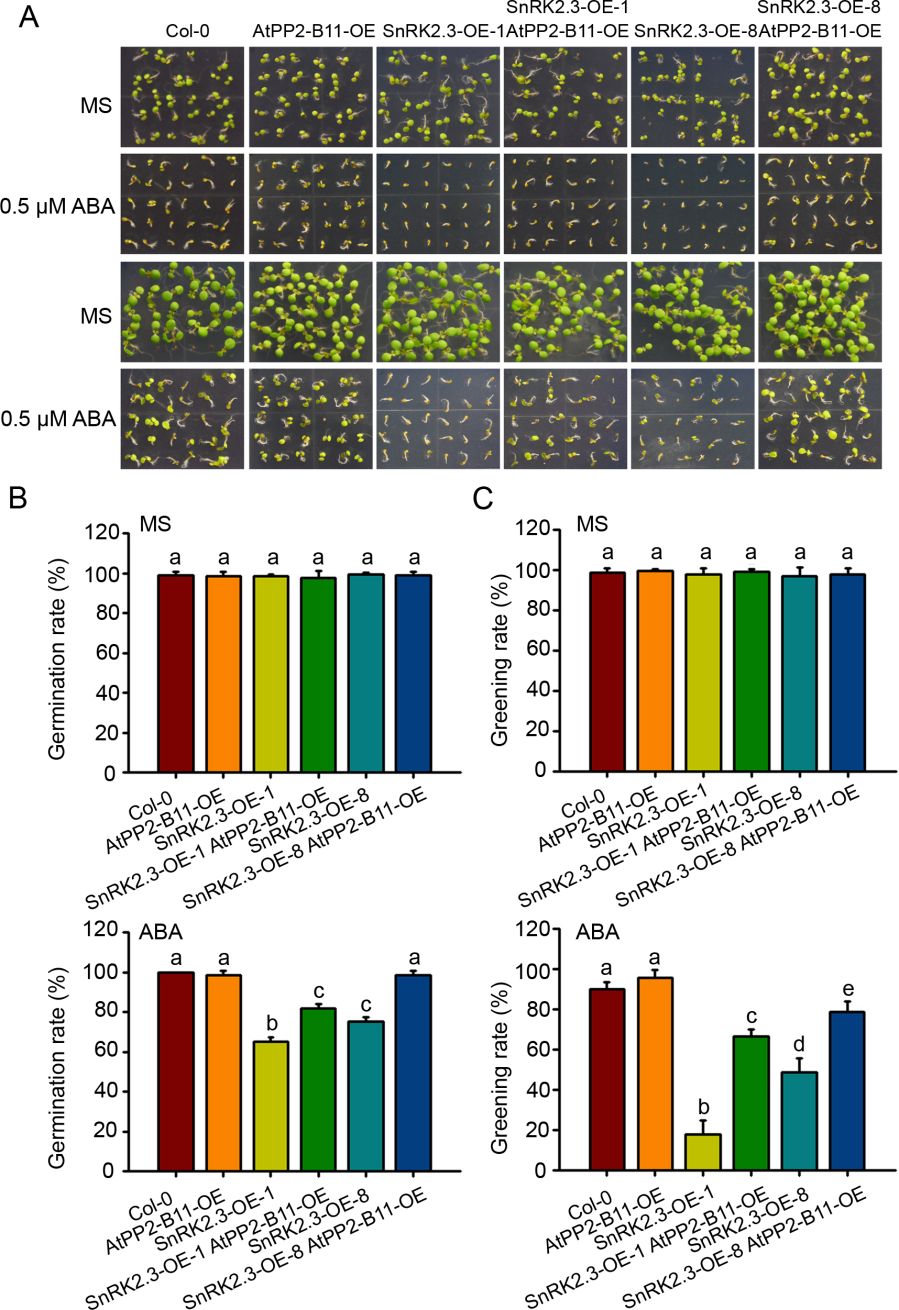
AtPP2-B11 inhibits the ABA sensitivity of *SnRK2*.*3* overexpression. (A). Phenotype assay of wild type, *AtPP2-B11-OE*, *AtPP2-B11-OE SnRK2*.*3-OE-1* and *AtPP2-B11-OE SnRK2*.*3-OE-8* overexpression transgenic lines in germination and greening stages with or without ABA. The image was taken at 5 days (top two panels) and 10 days (bottom two panels) after germination, respectively. (B). Comparision of germination rates between wild type, *AtPP2-B11-OE*, *AtPP2-B11-OE SnRK2*.*3-OE-1* and *AtPP2-B11-OE SnRK2*.*3-OE-8* at 5 days after stratification. (C). Comparision of greening rates between wild type, *AtPP2-B11-OE*, *AtPP2-B11-OE SnRK2*.*3-OE-1* and *AtPP2-B11-OE SnRK2*.*3-OE-8* at 10 days after germination. Different letters indicate a significant difference (Student-Newman–Kuels [SNK] test, P < 0.05).

SnRK2.3 is a positive regulator of ABA signaling pathway. Although the loss-of- function mutant of *SnRK2*.*3* has subtle ABA sensitivity [7], we found that the overexpression of *SnRK2*.*3* under the control of 35S promoter increased ABA sensitivity significantly. As shown in the Fig 8, *SnRK2*.*3-OE-1* and *SnRK2*.*3-OE-8* plants displayed hypersensitive phenotypes to ABA, and the ABA sensitivity of the transgenic plants was related to the protein level of SnRK2.3 in *SnRK2*.*3-OE-1* and *SnRK2*.*3-OE-8* plants. The result suggests a critical role of SnRK2.3 levels in mediating plant response to ABA; As AtPP2-B11 promotes the degradation of SnRK2.3, we predicted that overexpression of *AtPP2-B11* may alleviate the ABA hypersensitivity phenotype of SnRK2.3 overexpression plants. To test this possibility, we crossed *AtPP2-B11-OE* and *SnRK2*.*3-OE* to get the double overexpression line *AtPP2-B11-OE SnRK2*.*3-OE*. We then performed phenotype assay of Col-0, *SnRK2*.*3-OE-1*, *SnRK2*.*3-OE-8*, *AtPP2-B11-OE*, *AtPP2-B11-OE SnRK2*.*3-OE-1* and *AtPP2-B11-OE SnRK2*.*3-OE-8*. The result showed that the overexpression of *AtPP2-B11* in wild type background did not alter the ABA sensitivity of the plants; by contrast, overexpression of *AtPP2-B11* in the *SnRK2*.*3* overexpression background dramatically allevated the ABA hypersensitivity of *SnRK2*.*3* overexpression plants. In the presence of ABA, the germination rates of Col-0 and *AtPP2-B11-OE* were 100% *SnRK2*.*3-OE-1* and *SnRK2*.*3-OE-8* were 65% and 75%, respectively at 5 days after stratification. Intriguingly, the germination rates of the *AtPP2-B11-OE SnRK2*.*3-OE-1* and *AtPP2-B11-OE SnRK2*.*3-OE-8* double overexpression lines were up to 81.7% and 98.3%, respectively (Fig 8). The greening rates of Col-0 and *AtPP2-B11-OE* were 90% and 92%, *SnRK2*.*3-OE-1* and *SnRK2*.*3-OE-8* were 17.8% and 48.9%, respectively at 10 days after straitification, while the greening rates of the *AtPP2-B11-OE SnRK2*.*3-OE-1* and *AtPP2-B11-OE SnRK2*.*3-OE-8* double overexpression lines were up to 66.7% and 78.9%. The fact that the germination and greening rates of *AtPP2-B11-OE SnRK2*.*3-OE* double overexpression lines were much higher than that of *SnRK2*.*3-OE* lines indicates that *AtPP2-B11* can reduce the level of SnRK2.3 causing allevation of the ABA-mediated inhibition of seed germination and seedlings.

## Discussion

The ABA signaling pathway is composed of many important regulatory components, and dynamic regulation of these determinants governs both the signal transduction pathway and plant response to constantly changing environmental conditions. Upon perception, the ABA signal is relayed through protein-protein interactions; therefore, protein dynamics play a fundamental role in ABA-mediated cellular processes. Recently, the turnover of PYR1/PYLs, PP2Cs, and bZIP transcription factors has emerged as a key regulatory mechanism in the activation or attenuation of ABA signaling. SnRK2 kinases are essential regulators of ABA signaling that activate a set of bZIP transcription factors and ion transporters, thereby transducing the ABA signal and initiating cellular responses to ABA [6–8]. However, the regulation of SnRK2 turnover is poorly understood. In this study, we found that SnRK2.2/2.3/2.6 kinases undergo proteasome-mediated protein degradation; however, we also found that the F-box protein AtPP2-B11 interacts with SnRk2.2/SnRK2.3, but not SnRK2.6, and that it specifically targets SnRK2.3 for degradation via the SCF ubiquitin E3 ligase complex. Our results reveal for the first time the precise regulation of SnRK2 kinase turnover and add a new layer to the regulation of ABA signaling and plant stress responses.

Studies have shown that the genes *SnRK2*.*2/2*.*3/2*.*6* display different tissue- or cell-specific expression patterns [31]; however, they redundantly regulate ABA-mediated plant growth and stress tolerance, including seed germination, cotyledon greening, seedling growth, stomatal closure, and drought tolerance [6–8]. *SnRK2*.*2/2*.*3/2*.*6* are differentially regulated at the transcriptional level, although the detailed mechanisms underlying that regulation are unknown. Structural analyses have shown that the ABA-induced release of PP2C inhibition enables SnRK2 kinases to become active and phosphorylated; however, the efficiency of SnRK2 phosphorylation varies somewhat for different kinases [3, 8–15], implying that the activities of these proteins in terms of the activation of downstream effectors may be different. In this study, we found that SnRK2.2, SnRK2.3, and SnRK2.6 underwent 26S proteasome-mediated degradation by using MG132, a proteasome inhibitor (Fig 1). Notably, we demonstrated that F-box protein AtPP2-B11 promotes the degradation of SnRK2.3 specifically. Four lines of evidence support this notion. First, our protein-protein interaction assays revealed that the F-box protein AtPP2-B11, a substrate receptor for the SCF E3 ligase complex, interacts specifically with SnRK2.3 but not SnRK2.2 or SnRK2.6 *in vivo* (Fig 2). Second, the overexpression of AtPP2-B11 promoted the degradation of SnRK2.3, but not SnRK2.2 or SnRK2.6, respectively (Fig 3 and S11 Fig). Third, the expression pattern of *AtPP2-B11* treated by ABA is similar to that of SnRK2.3 [31] (Fig 5B). Fourth, both the knockdown and knockout of *AtPP2-B11* and overexpression of *SnRK2*.*3* increases ABA sensitivity in seed germination and postgermination stages, and overexpression of *AtPP2-B11* can allevate the ABA hypersensitive phenotype to ABA of *SnRK2*.*3-OE* plants (Fig 6; Fig 8; S14 Fig). However, we still do not know why these highly conserved SnRK2s have different binding specificities for AtPP2-B11.

SCF ubiquitin E3 ligases have strong substrate specificity, and F-box proteins belonging to the SCF ubiquitin protein degradation system determine the substrate specificity [15–17]. Therefore, it is possible that these SnRK2s are targeted by different F-box proteins. Here, we found that AtPP2-B11 is a component of the SCF ubiquitin E3 ligase complex; it associated physically with ASK1 and ASK2 (S5 Fig), consistent with previous results [32]. Most importantly, we proved that AtPP2-B11 negatively regulates ABA-mediated processes by specifically targeting SnRK2.3 for degradation (Fig 1; Fig 3; S10 Fig; S11 Fig), and the degradation of SnRK2.3 is induced by ABA *in vitro* and *in vivo* (Fig 4). *AtPP2-B11* encodes an F-box protein, and previous reports have shown that *AtPP2-B11* is responsive to abiotic stress and is involved in plant tolerance to drought and salt stress [29, 30]. In this study, we showed that *AtPP2-B11* was expressed in multiple tissues and organs, and that it was induced by ABA (Fig 5). In particular, *AtPP2-B11* was highly expressed in cotyledons and hypocotyl of germinating seedlings regardless of ABA treatment, suggesting a critical role of the gene in early developmental stage. However, we could not detect any expression of *AtPP2-B11* in vascular tissues of root, leaf and stem of plants, except the cotyledons of germinating seedlings. This is unexpected because *AtPP2-B11* encodes a phloem protein which is a member of phloem protein 2 family. In *Arabidopsis*, PP2 family contains 30 members belonging to two subgroups PP2-A and PP2-B. Up to date, only *PP2-A1* and PP2-A2 have been shown to be highly expressed in phloem [36]. It is possible that *AtPP2-B11* is expressed at relative low level in phloem. This notion was supported by the eFP expression data because low levels of *AtPP2-B11* expression were detected in phloem companion cells of *Arabidopsis* root. It is reasonable if *AtPP2-B11* is expressed in phloem companion cells because ABA is synthesized in phloem companion cells and can be transported to other cells and tissues to induce plant response to ABA and abiotic stresses [37]. Based on the expression pattern, it is highly likely that AtPP2-B11 mediates plant responses to ABA in multiple tissues, especially seed germination and early seedling growth and reproductive organs in Arabidopsis.

**Fig 9:**
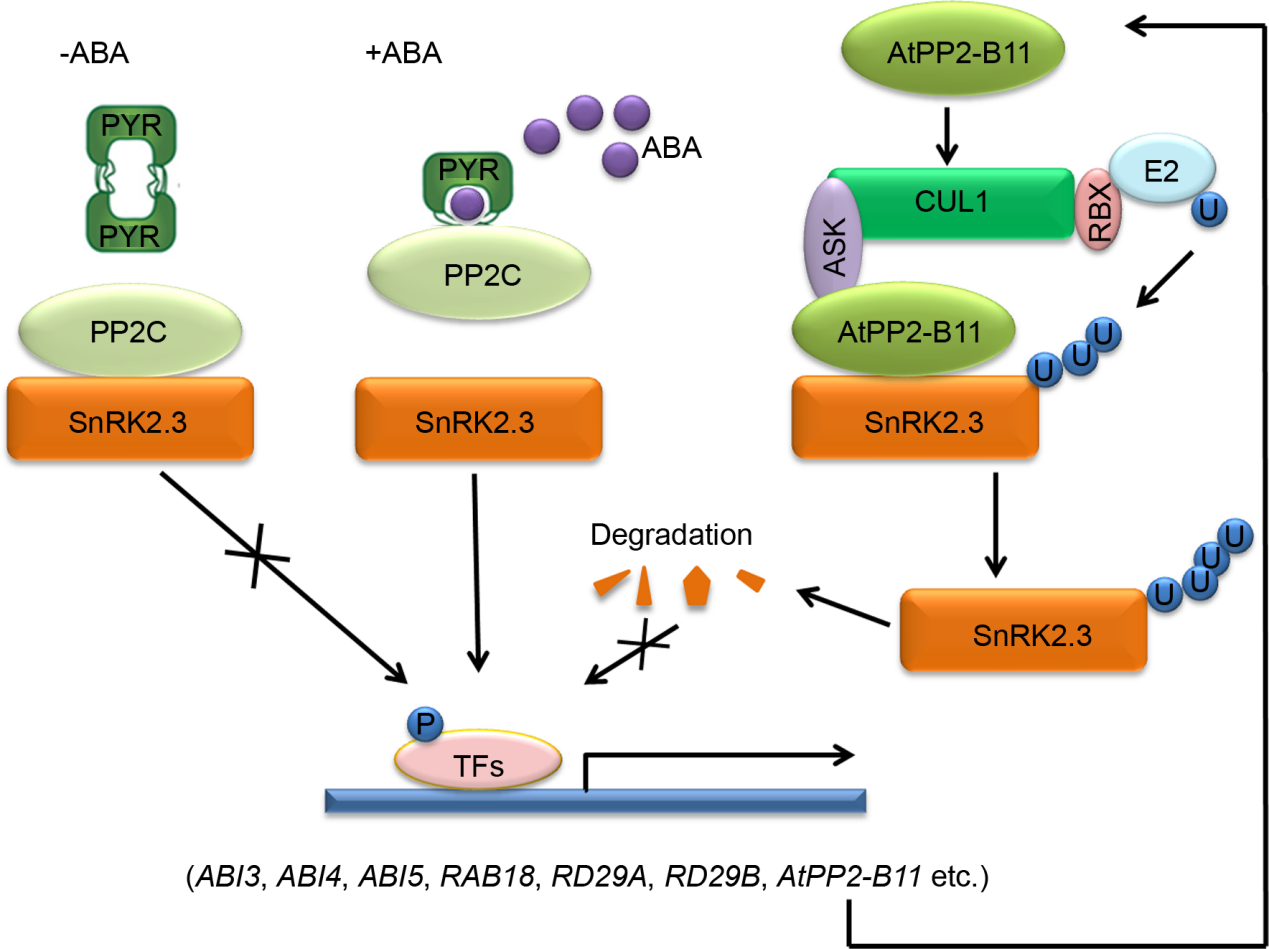
A proposed model for SnRK2.3 degradation. In the absence of ABA, PP2C interacts with SnRK2.3, inhibiting its kinase activity and preventing the phosphorylation of downstream transcription factors by SnRK2.3. However, in the presence of ABA, PP2C binds to PYR/PYL/RCAR proteins and ABA, thereby inhibiting PP2C phosphatase activity. SnRK2.3 is then activated to phosphorylate transcriptional factors and induce the expression of downstream genes, including *AtPP2-B11*. AtPP2-B11, the substrate receptor, interacts with ASK to form SCF E3 ligase complex. AtPP2-B11 specifically targets SnRK2.3 for its degradation to turn off ABA signaling.

Our functional analyses results demonstrated that *AtPP2-B11* plays an important role in plant ABA responses. The knockdown and knockout of *AtPP2-B11* dramatically increased plant sensitivity to ABA during seed germination and seedling establishment (Fig 6; S14 Fig). It is clear that AtPP2-B11 is required for *Arabidopsis* plants to properly respond to ABA and that it may protect plants from overreacting to ABA. Thus, we successfully identified a specific substrate receptor for SnRK2.3 turnover via SCF ubiquitin proteasome-mediated degradation that is crucial for the attenuation of ABA signaling and for an ABA-dependent plant growth arrest (Fig 9). We didn’t observe obvious phenotype of overexpression of *AtPP2-B11* in response to ABA, this is because SnRK2.2, SnRK2.3 and SnRK2.6 are redundant in regulating seed germination and plant growth. This is consistent with that the single mutant of *snrk2*.*3* mutant exhibited subtle phenotypes in terms of its ABA response and drought tolerance [6]. Since *AtPP2-B11* is expressed in multiple tissues at different developmental stages, we predicted that *AtPP2-B11* is also involved in plant response to ABA in these tissues. Further phenotypic analyses of the knockdown and knockout of *AtPP2-B11* mutants will provide useful information about the role of the gene.

Despite the specific role of AtPP2-B11 in facilitating SnRK2.3 degradation, some important questions remain. We still do not know how the degradation of SnRK2.3 is triggered. A recent study showed that casein kinase 2 (CK2) triggers the proteasome-mediated degradation of SnRK2.6 by phosphorylating the ABA box of SnRK2.6 and enhancing its binding activity to PP2Cs in maize [13]. It was proposed that CK2 plays the same role in facilitating the degradation of SnRK2.2 and SnRK2.3 in maize, but direct evidence is lacking. Taking into account that SnRK2s are highly conserved in plants, it will be interesting to test whether CK2 mediates the phosphorylation of SnRK2.3 at the ABA box and facilitates the SnRK2.3-PP2C interaction in *Arabidopsis*. The modification of SnRK2s and their ability to interact with PP2Cs could significantly affect the protein stability of these kinases. Further investigation will facilitate our understanding of the molecular mechanism through which SnRK2s and the core ABA regulatory signaling module are dynamically and precisely regulated. This will no doubt help us decipher the mechanisms underlying plant tolerance to abiotic stresses.

## Materials and methods

### Plant materials and growth conditions

*Arabidopsis thaliana* ecotype Col-0 was used in this study. The *atpp2-b11* (GK-162G12) T-DNA insertion mutant was obtained from the Nottingham *Arabidopsis* Stock Centre (http://arabidopsis.info/). The primers used for homozygous analysis of T-DNA insertion mutant are shown in S1 Table. Seeds were surface-sterilized with 50% bleach and washed three to four times with sterile water. The seeds were kept at 4°C in darkness for 2–3 days. The seeds were germinated and grown on MS medium (Sigma-Aldrich, St. Louis, MO, USA) containing 1% (w/v) sucrose and 0.3% Phytogel (Sigma-Aldrich). The percentage of germinated seeds and percentage of seedlings with green cotyledons were recorded at the specified time points. Two-week-old seedlings were transplanted to soil and grown under a 16-h light/8-h dark photoperiod at 22°C.

### Phenotypic analysis assay

For phenotypic analysis in the presence of ABA, MS medium was prepared supplemented with 1% sucrose and different concentrations of ABA. Seeds were surface-sterilized and sowed on the MS medium with ABA or not (three plates per treatment). The plates were stratified at 4°C for 2 days and then transferred to chamber at 22°C. Seedlings (30 seedlings per line) with elongated radicles or with green cotyledons were counted at the indicated time points.

### Promoter analysis

Sequence of a 831bp-fragment upstream of *AtPP2-B11* was analyzed using PlantCARE (http://bioinformatics.psb.ugent.be/webtools/plantcare/html/).

### Yeast two-hybrid and BiFC analyses

The constructs were created in two pairs of Gateway-compatible destination vectors: pGBKT7 (binding domain [BD]) with pGADT7 (activation domain [AD]) and pEarleyGate201-YN (N-terminal YFP) with pEarleyGate202-YC (C-terminal YFP). The coding sequences of AtPP2-B11, ASK1, ASK2, and the SnRK2s were amplified from Col-0 cDNA using the specified primer pairs, inserted into pDONOR207, and then recombined in the appropriate destination vector. For the yeast two-hybrid assays, yeast strain AH109 was used for co-transformation of the AD and BD constructs. Aliquots (5 μl each) of the diluted co-transformed AH109 culture were spotted onto SD plates lacking Trp and Leu or lacking Trp, Leu, His, and Ade, and incubated at 30°C for 3–5 days to observe yeast growth. Plasmids AD-T and BD-53 were used as a positive control, while the AD and BD were used as a negative control. For the BiFC assays, *Agrobacterium tumefaciens* carrying the YFP N-terminal and YFP C-terminal fusion constructs were infiltrated into *N*. *benthamiana* leaves. Reconstituted YFP signals were observed using confocal imaging 36–48 h after infiltration.

### GUS histochemical analysis

For our GUS assays, an 813-bp fragment upstream of the ATG was cloned into pCAMBIA1391 to generate *pAtPP2-B11*::*GUS*. At different time points, seeds, seedlings, leaves, stems, inflorescences, siliques, and roots were harvested and treated with MS with or without 50 μM ABA for 3 h, and then incubated in a freshly prepared buffer containing 5-bromo-4-chloro-3-indolyl-b-D-glucuronic acid for the specified time at 37°C in the dark, followed by clearance with 75% ethanol.

### Real time PCR analysis of gene expression

Total RNA was extracted from 7-day-old and 10-day-old *Arabidopsis* seedlings using TRIzol reagent. Reverse transcription was then performed using a cDNA synthesis Supermix with gDNA remover (Transgen Biotech, China). Quantitative real-time PCR was run using the ABI Prime 7500 Sequence Detection System with Platinum SYBR Green qPCR Supermix-UDG (Invitrogen, Carlsbad, CA, USA). The transcript abundance was normalized against the reference gene *ACTIN2* and *UBC5*. The experiments were performed three times, each with three replicates.

### Cell-free and *in vivo* protein degradation assays

To purify the recombinant proteins MBP-SnRK2.2, MBP-SnRK2.3, and MBP-SnRK2.6 in *E*. *coli*, the open reading frames (ORFs) of *SnRK2*.*2*, *SnRK2*.*3*, and *SnRK2*.*6* were cloned into pMAL-c2x. The resulting plasmids were then transformed into the expression strain BL21. Protein purification was performed using Amylose Resin (NEB, UK) according to the manufacturer’s instructions. The cell-free assay was performed as described. Proteins were extracted using a buffer containing 50mM Tris-MES (pH 8.0), 0.5 M sucrose, 1mM MgCl2, 10mM EDTA (pH 8.0), 5mM DTT from 7-day-old seedlings [23]. Next, 200 μg of plant protein were incubated with 200 ng of purified protein in a buffer containing 50 mM Tris-MES (pH 8.0), 0.5 M sucrose, 1 mM MgCl2, 10 mM EDTA (pH 8.0), 5 mM DTT at 22°C for the indicated time. MG132 was used as a specific inhibitor of 26S proteasome mediated degradation. The MBP tagged protein was detected by the MBP antibody (MBP Tag Antibody; catalog number: 66003-1-lg; proteintech).

For the *in vivo* protein degradation experiment, proteins were extracted from 7-day-old seedlings with the treatment of 50 μM MG132 or not for indicated time using a buffer containing 50 mM Tris-MES (pH 8.0), 0.5 M sucrose, 1 mM MgCl2, 10 mM EDTA (pH 8.0), 5 mM DTT. 2×SDS loading buffer was added to the samples. The samples were boiled and then tested with anti-Flag (Monoclonal ANTI-FLAG M2-Peroxidase (HRP) antibody produced in mouse; Sigma).

### Pull-down and Co-IP assays

To purify recombinant GST-AtPP2-B11, MBP-SnRK2.2, MBP-SnRK2.3, and MBP-SnRK2.6 in *E*. *coli*, the ORFs of *AtPP2-B11* and the SnRK2 genes were cloned into pGEX-4T-1 and pMAL-c2x. The GST-AtPP2-B11 and MBP-SnRK2s plasmids were then transformed into the expression strain BL21. Protein purification was performed using Glutathione Agarose (ThermoFisher Science, USA) and Amylose Resin (NEB, UK) according to the manufacturers’ instructions.

GST beads were cleaned with GST binding buffer four times to remove the ethanol. The purified proteins GST and AtPP2-B11-GST were then incubated with the same volume of GST beads in GST binding buffer for 2 h at room temperature, washed with GST binding buffer four times to remove redundant proteins, and incubated with MBP-SnRK2s for another 2 h at room temperature or 4°C overnight. Next, the cultures were washed four times to remove redundant MBP-SnRK2s. Samples were then collected, mixed with 2× SDS protein loading buffer, and boiled for 5 min for western blotting. Anti-MBP (MBP Tag Antibody; catalog number: 66003-1-lg; proteintech) and anti-GST (*ProteinFind* Anti-GST Mouse Monoclonal Antibody; Transgen Biotech) were used to detected the MBP and GST tagged proteins.

For the Co-IP experiments, *Arabidopsis* seedlings carrying *Pro35S*::*AtPP2-B11-Myc* and *Pro35S*::*SnRK2*.*2-Flag* or *Pro35S*::*SnRK2*.*3-Flag* or *Pro35S*::*SnRK2*.*6-Flag* were used. Ten-day-old seedlings were homogenized in protein extraction buffer (50 mM Na_2_HPO_4_/NaH_2_PO_4_ [pH 7.4], 150 mM NaCl, 1% Triton X-100, 15% glycerol, 1 mM phenylmethylsulfonyl fluoride [PMSF], and protease inhibitor cocktail [Roche, Basel, Switzerland]). After protein extraction, flag beads (ANTI-FLAG M2 Affinity Gel; Sigma-Aldrich) were washed four times with phosphate-buffered saline and incubated with the extracted proteins at 4°C for 2 h or room temperature for 1 h. The precipitated samples were washed at least four times with the protein wash buffer (50 mM Na_2_HPO_4_/NaH_2_PO_4_ [pH 7.4], 150 mM NaCl, 0.1% Triton X-100, 10% glycerol, 1 mM PMSF, and protease inhibitor cocktail [Roche]) and then eluted with 2× SDS protein loading buffer and boiled for 5 min for western blotting. The Flag-tag and Myc-tag proteins were detedted by Flag antibody (Monoclonal ANTI-FLAG M2-Peroxidase (HRP) antibody produced in mouse; Sigma) and Myc antibody (Anti-c-Myc antibody produced in rabbit; Sigma).

Protoplasts generated from the leaves of wild type Col-0 were transformed with transient expression plasmids as described [38]. After 8 h incubation at room tempreture, followed by 50 μM MG132 treatment for 1h and then treatment with or without 20 μM ABA for another 2 h. Protoplasts were collected in protein extraction buffer (10 mM HEPES (pH = 7.5), 100 mM NaCl, 1 mM EDTA, 10% Glycerol, 0.5% Triton X-100 and protease inhibitor cocktail). The lysate was centrifuged at 15,000g for 10 min at 4°C, and the supernatants were incubated with HA antibody for 2 h at 4°C, and then incubated with Protein A Magnetic beads (*BIO-RAD*) for another 2 h at 4°C. The beads were washed three times with wash buffer (10 mM HEPES (pH = 7.5), 100 mM NaCl, 1 mM EDTA, 10% Glycerol, 0.1% Triton X-100 and protease inhibitor cocktail), and added 2 x protein loading buffer for Western blot assay, using anti-Flag antibody to detect the ubiquitination levels and to detect the SnRK2.3-HA using anti-HA antibody.

### Statistical analysis

All data were analyzed using SigmaPlot 10.0 (Systat Software, Inc., Chicago, IL). The averages and standard deviations of all results were calculated, and Student’s t-tests were performed to generate P values.

## Supporting information

S1 Fig SnRK2.3 protein expression level analysis in *SnRK2*.*3-OE-1* and *SnRK2*.*3-OE-8* transgenic seedlings. Proteins were extracted from 7-day-old transgenic seedlings *SnRK2*.*3-OE-1* and *SnRK2*.*3-OE-8* grown on MS medium. The SnRK2.3 protein level was checked by western blotting using anti-Flag antibody. Ponceau staining was used as loading control.
(TIF)

S2 Fig Assay for the negative control of the interaction with AtPP2-B11 and SnRK2.2, SnRK2.3 or SnRK2.6. AtPP2-B11 fused with N-terminal YFP was coexpressed with empty C-terminal YFP and SnRK2s fused with C-terminal YFP was coexpressed with empty N-terminal YFP in *Nicotiana benthamiana* leaves. the YFP signal was observed using a Leica confocal laser scanning microscope at 36 h after infiltration.
(TIF)

S3 Fig The protein structure of AtPP2-B11. *AtPP2-B11* encodes a protein contains 257 aa with a F-box domain in the N terminal and a PP2 domain in its C terminal.
(TIF)

S4 Fig AtPP2-B11 is localized in both nucleus and cytoplasm. Subcellular localization of AtPP2-B11-GFP and GFP in tabacoo leaf cells (A) and *Arabidopsis* transgenetic lines (B). The *35S*::*GFP-AtPP2-B11* and *35S*::*GFP* constructs were transfected into the tabacoo leaves and the GFP fluorescence was observed 36 h after infiltration using a fluorescence microscope. DAPI (4’, 6-diamidino-2-phenylindole) staining indicated the nucleus (top panel). The *35S*::*GFP-AtPP2-B11* construct was transfected into *Arabidopsis*. GFP fluorescence was detected in the roots of transgenic *35S*::*GFP-AtPP2-B11* plants (bottom panel). GFP-AtPP2-B11 fusion protein was extracted from *Arabidopsis* transgenic lines and AtPP2-B11 was detected by anti-GFP antibody.
(TIF)

S5 Fig AtPP2-B11 interacts with ASK1 and ASK2. (A). Interaction assays were conducted for AtPP2-B11 and ASK1/ASK2. AH109 cells that coexpressed AtPP2-B11 with ASK1 or ASK2 were grown on synthetic dropout medium lacking tryptophan and leucine (-WL) and synthetic dropout medium lacking tryptophan, leucine, histidine and adenine (-WLHA). Saturated cultures were spotted onto -WLHA medium at different dilutions (OD_600_ = 1, 10^−1^, 10^−2^, 10^−3^, and 10^−4^). The vectors AD-T and BD-53 were used as positive controls; the empty vectors pGADT7 (AD) and pGBKT7 (BD) were used as negative controls. (B). BiFC assays between AtPP2-B11 and ASK1/ASK2. AtPP2-B11-YFP^N^ and ASK1 -YFP^C^ or ASK2-YFP^C^ were coexpressed in *N*. *benthamiana*. The YFP signal (left), brightfield images (middle), and merged images (right) are shown.
(TIF)

S6 Fig The negative control of the interaction of AtPP2-B11 with ASK1 and ASK2. AtPP2-B11 fused with N-terminal YFP was coexpressed with empty C-terminal YFP and ASK1/2 fused with C-terminal YFP was coexpressed with empty N-terminal YFP in *Nicotiana benthamiana* leaves. The YFP signal was observed using a Leica confocal laser scanning microscope at 36 h after infiltration.
(TIF)

S7 Fig The gene transcript abundance of *AtPP2-B11* mutants and the phenotype compared with wild type in normal conditions. (A). The gene transcript abundance of overexpression of *AtPP2-B11*, RNA was extracted from the 7-day-old seedlings grown on MS medium, three independent experiments were performed with similar results, each with three replicates. The student’s t-test was performed and the statically significant treatments were marked with ‘***’ (P<0.001). (B). The gene transcript abundance of the knock down mutants of *AtPP2-B11*, RNA was extracted from the 7-day-old seedlings with or without 50 μM ABA treatment for 3 hours, three independent experiments were performed with similar results, each with three replicates. The student’s t-test was performed and the statically significant treatments were marked with ‘***’ (P<0.001). (C). 3-week-old (top) and 5-week-old (bottom) seedlings of Col-0, *AtPP2-B11* amiRNA lines *(amiR7* and *amiR15)* and overexpression line (*OE*). Bar = 5 cm. (D). 2-month-old plants of Col-0, *AtPP2-B11* amiRNA lines *(amiR7* and *amiR15)* and overexpression line (*OE*). Bar = 5 cm.
(TIF)

S8 Fig The transcript abundance of *AtPP2-B11* in *35S*::*SnRK2*.*3-3flag*/Col-0 and *35S*::*SnRK2*.*3-3flag*/*AtPP2-B11*. RNA was extracted from 7-day-old seedlings and the transcript abundance was normalized to *UBC5*. The student’s t-test was performed and the statically significant treatments were marked with ‘***’ (P<0.001).
(TIF)

S9 Fig Identification of the mutant *atpp2-b11*. (A). The genomic structure and T-DNA insertions in *AtPP2-B11*. Exons are depicted as blue boxes, introns are represented by black lines, and 5’and 3’UTR are represented by green boxes, black box represents the promoter region and the black triangle represents the T-DNA insertion site. The arrows represent the primers sites. (B). Identification of homozygous of *atpp2-b11*. (C). Transcription assay of *AtPP2-B11* in Col-0 and *atpp2-b11* mutant.
(TIF)

S10 Fig The cell-free degradation of SnRK2.3 in WT and *atpp2-b11* seedlings. Cell-free assays of SnRK2.3-MBP degradation by incubation of SnRK2.3-MBP with ABA pre-treatment protein extracts from WT or *atpp2-b11* knockout mutant. Ponceau staining was used as loading control. Relative amounts of proteins were determined by ImageJ and normalized to loadings determined by Ponceau staining and expressed relative to the value at 0 hr time. Different letters indicate a significant difference (Student-Newman–Kuels [SNK] test, P < 0.05). Quantitative analysis of the band intensity was on the right side of the figure. Error bars are means ± s.e.m. (n ≥ 3 independent experiments).
(TIF)

S11 Fig The degradation of SnRK2.2 and SnRK2.6. (A). and (B). Cell free degradation of SnRK2.2 and SnRK2.6. Proteins were extracted from 7-day-old seedlings of wild type or *AtPP2-B11* overexpression transgenic lines. Ponceau staining was used as loading control. Relative amounts of proteins were determined by ImageJ and normalized to loadings determined by Ponceau staining and expressed relative to the value at 0 hr time. Different letters indicate a significant difference (Student-Newman–Kuels [SNK] test, P < 0.05). Quantitative analysis of the band intensity was on the right side of the figure. Error bars are means ± s.e.m. (n ≥ 3 independent experiments). (C). and (D). Cell free degradation of SnRK2.2 and SnRK2.6. Proteins were extracted from 7-day-old seedlings of wild type or *AtPP2-B11* amiRNA knock down line (*amiR15*). The *amiR15* and wild type seedlings were pre-treatment with 50 μM ABA for 5 h. Ponceau staining was used as loading control. Proteins were detected as in (A and B). Different letters indicate a significant difference (Student-Newman–Kuels [SNK] test, P < 0.05). Quantitative analysis of the band intensity was on the right side of the figure. Error bars are means ± s.e.m. (n ≥ 3 independent experiments).
(TIF)

S12 Fig The expression pattern of *AtPP2-B11*. The expression pattern of *AtPP2-B11* in response to ABA from the microarray data of public available source (TAIR).
(TIF)

S13 Fig Promoter analysis of *AtPP2-B11*. The 831bp DNA fragment upstream of the ATG staring code of the *AtPP2-B11* was analyzed using PlantCARE (http://bioinformatics.psb.ugent.be/webtools/plantcare/html/).
(TIF)

S14 Fig The phenotype analysis of *atpp2-b11*. (A) Phenotypic analyses of wild-type (Col-0) and *atpp2-b11* treated with 0.5 μM ABA. The images were taken after 4 and 8 days, respectively. (B) The analysis of germination rate and greening rate.
(TIF)

S15 Fig The transcript abundance of ABA-responsive genes in Col-0 and *atpp2-b11*. The relative transcript abundance of (A) *ABI3*, (B) *ABI4*, (C) *ABI5*, (D) *RAB18*, (E) *RD29A*, and (F) *RD29B* in Col-0 and *atpp2-b11* mutant plants were analyzed. Seedlings were grown on MS medium with or without 0.5 μM ABA for 7 days. Three independent experiments were performed with similar results, each with three replicates. *UBC5* was used as the internal control. The student’s t-test was performed and the statically significant treatments were marked with ‘***’ (P < 0.001) and ‘**’ (P < 0.01).
(TIF)

S1 Table The primers used in this study. (PDF)
