# Supplementary figures and images for: SCFAtPP2-B11 modulates ABA signaling by facilitating SnRK2.3 degradation in Arabidopsis thaliana
Source: PLoS Genet. 2017 Aug 7;13(8):e1006947. doi: 10.1371/journal.pgen.1006947 (PMC5560758; doi:10.1371/journal.pgen.1006947)

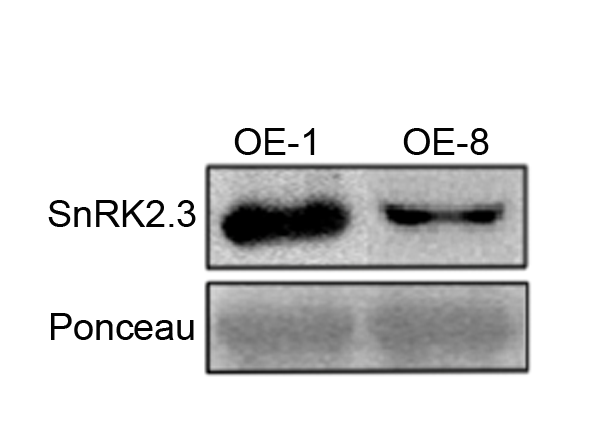

Supplement: S1 Fig — Proteins were extracted from 7-day-old transgenic seedlings SnRK2.3-OE-1 and SnRK2.3-OE-8 grown on MS medium. The SnRK2.3 protein level was checked by western blotting using anti-Flag antibody. Ponceau staining was used as loading control. (TIF) [file pgen.1006947.s001.tif]

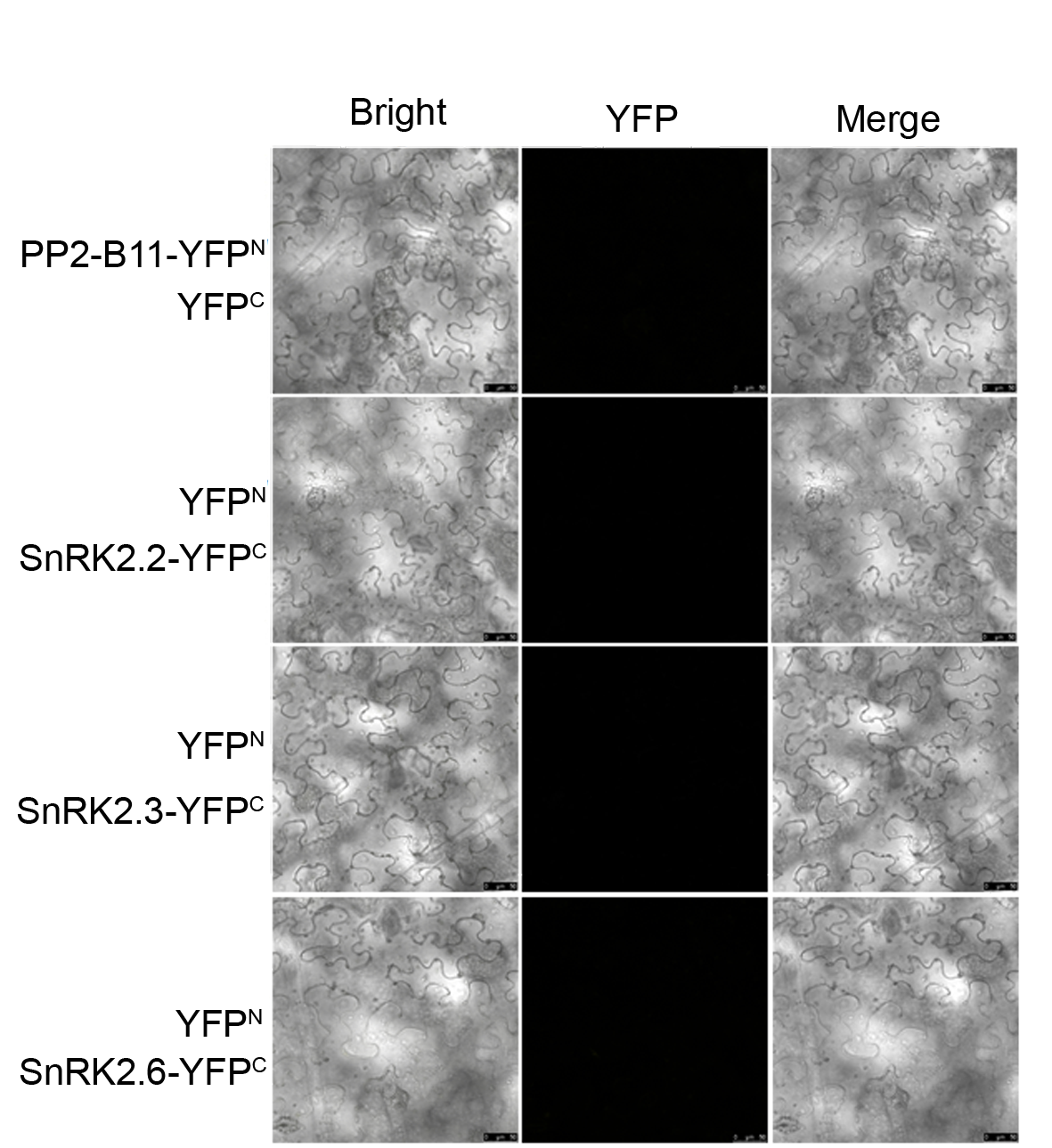

Supplement: S2 Fig — AtPP2-B11 fused with N-terminal YFP was coexpressed with empty C-terminal YFP and SnRK2s fused with C-terminal YFP was coexpressed with empty N-terminal YFP in Nicotiana benthamiana leaves. the YFP signal was observed using a Leica confocal laser scanning microscope at 36 h after infiltration. (TIF) [file pgen.1006947.s002.tif]

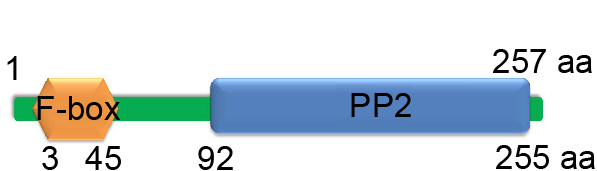

Supplement: S3 Fig — AtPP2-B11 encodes a protein contains 257 aa with a F-box domain in the N terminal and a PP2 domain in its C terminal. (TIF) [file pgen.1006947.s003.tif]

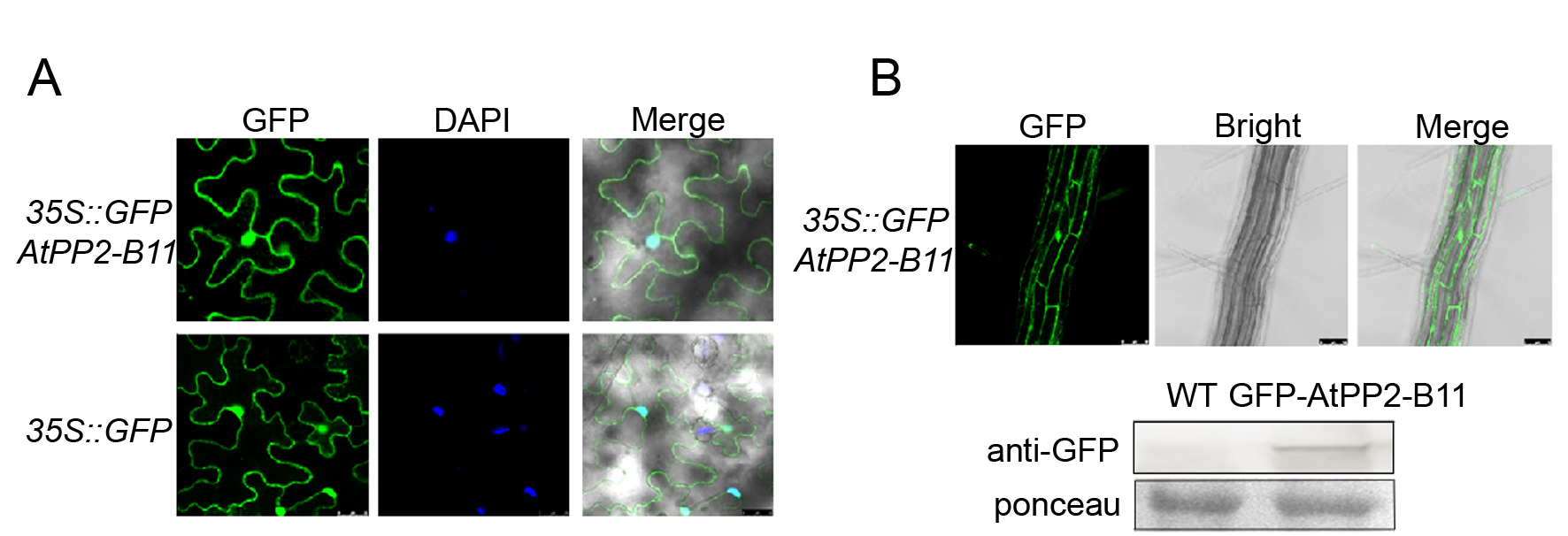

Supplement: S4 Fig — Subcellular localization of AtPP2-B11-GFP and GFP in tabacoo leaf cells (A) and Arabidopsis transgenetic lines (B). The 35S::GFP-AtPP2-B11 and 35S::GFP constructs were transfected into the tabacoo leaves and the GFP fluorescence was observed 36 h after infiltration using a fluorescence microscope. DAPI (4’, 6-diamidino-2-phenylindole) staining indicated the nucleus (top panel). The 35S::GFP-AtPP2-B11 construct was transfected into Arabidopsis. GFP fluorescence was detected in the roots of transgenic 35S::GFP-AtPP2-B11 plants (bottom panel). GFP-AtPP2-B11 fusion protein was extracted from Arabidopsis transgenic lines and AtPP2-B11 was detected by anti-GFP antibody. (TIF) [file pgen.1006947.s004.tif]

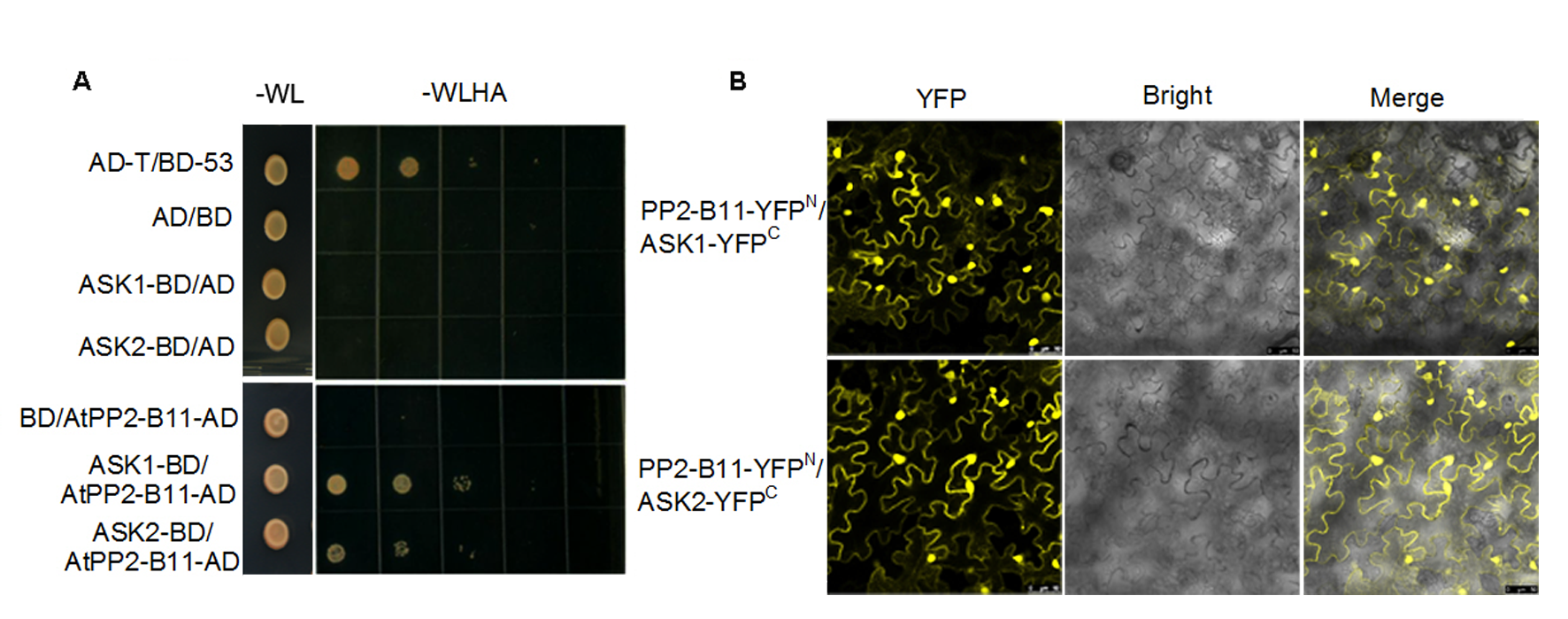

Supplement: S5 Fig — (A). Interaction assays were conducted for AtPP2-B11 and ASK1/ASK2. AH109 cells that coexpressed AtPP2-B11 with ASK1 or ASK2 were grown on synthetic dropout medium lacking tryptophan and leucine (-WL) and synthetic dropout medium lacking tryptophan, leucine, histidine and adenine (-WLHA). Saturated cultures were spotted onto -WLHA medium at different dilutions (OD600 = 1, 10−1, 10−2, 10−3, and 10−4). The vectors AD-T and BD-53 were used as positive controls; the empty vectors pGADT7 (AD) and pGBKT7 (BD) were used as negative controls. (B). BiFC assays between AtPP2-B11 and ASK1/ASK2. AtPP2-B11-YFPN and ASK1 -YFPC or ASK2-YFPC were coexpressed in N. benthamiana. The YFP signal (left), brightfield images (middle), and merged images (right) are shown. (TIF) [file pgen.1006947.s005.tif]

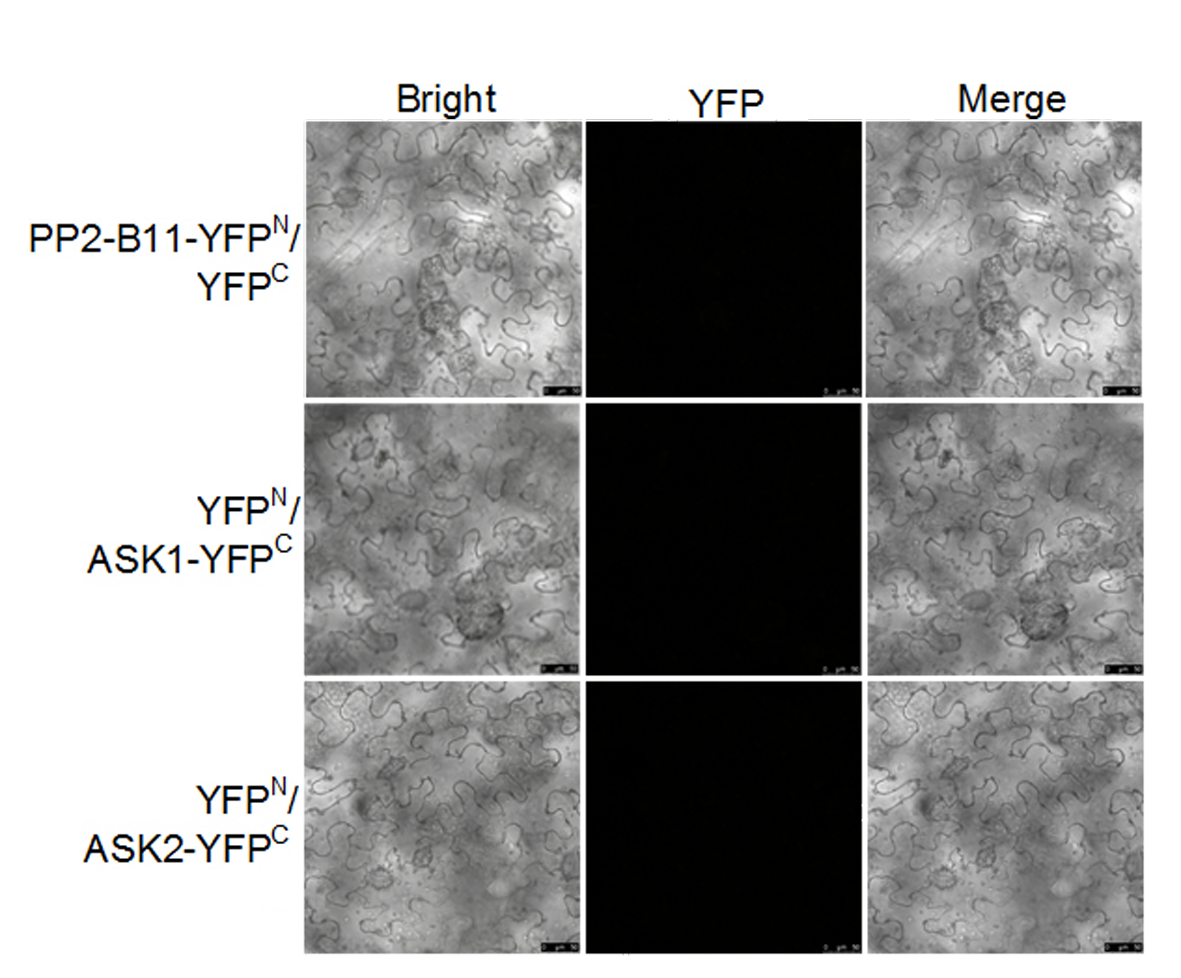

Supplement: S6 Fig — AtPP2-B11 fused with N-terminal YFP was coexpressed with empty C-terminal YFP and ASK1/2 fused with C-terminal YFP was coexpressed with empty N-terminal YFP in Nicotiana benthamiana leaves. The YFP signal was observed using a Leica confocal laser scanning microscope at 36 h after infiltration. (TIF) [file pgen.1006947.s006.tif]

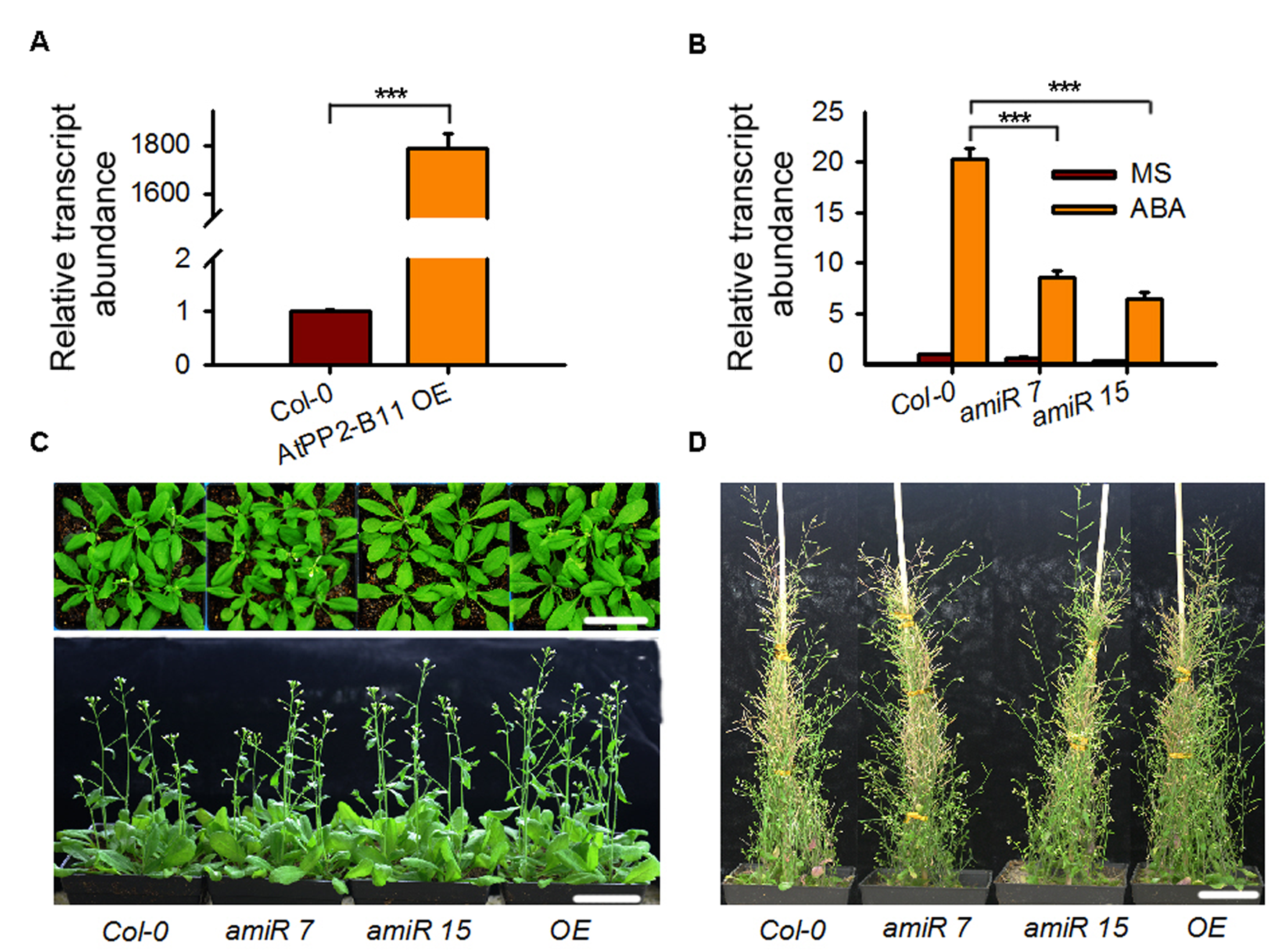

Supplement: S7 Fig — (A). The gene transcript abundance of overexpression of AtPP2-B11, RNA was extracted from the 7-day-old seedlings grown on MS medium, three independent experiments were performed with similar results, each with three replicates. The student’s t-test was performed and the statically significant treatments were marked with ‘***’ (P<0.001). (B). The gene transcript abundance of the knock down mutants of AtPP2-B11, RNA was extracted from the 7-day-old seedlings with or without 50 μM ABA treatment for 3 hours, three independent experiments were performed with similar results, each with three replicates. The student’s t-test was performed and the statically significant treatments were marked with ‘***’ (P<0.001). (C). 3-week-old (top) and 5-week-old (bottom) seedlings of Col-0, AtPP2-B11 amiRNA lines (amiR7 and amiR15) and overexpression line (OE). Bar = 5 cm. (D). 2-month-old plants of Col-0, AtPP2-B11 amiRNA lines (amiR7 and amiR15) and overexpression line (OE). Bar = 5 cm. (TIF) [file pgen.1006947.s007.tif]

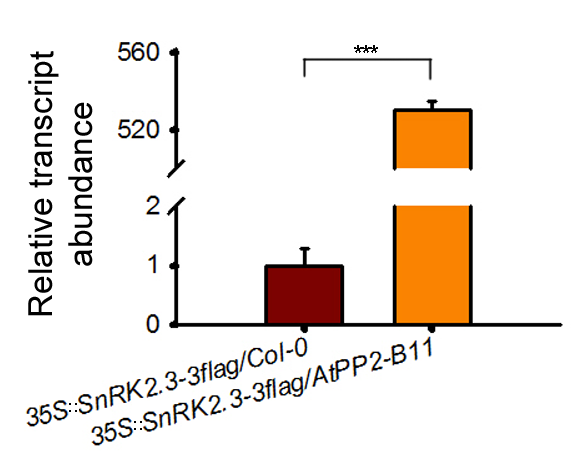

Supplement: S8 Fig — RNA was extracted from 7-day-old seedlings and the transcript abundance was normalized to UBC5. The student’s t-test was performed and the statically significant treatments were marked with ‘***’ (P<0.001). (TIF) [file pgen.1006947.s008.tif]

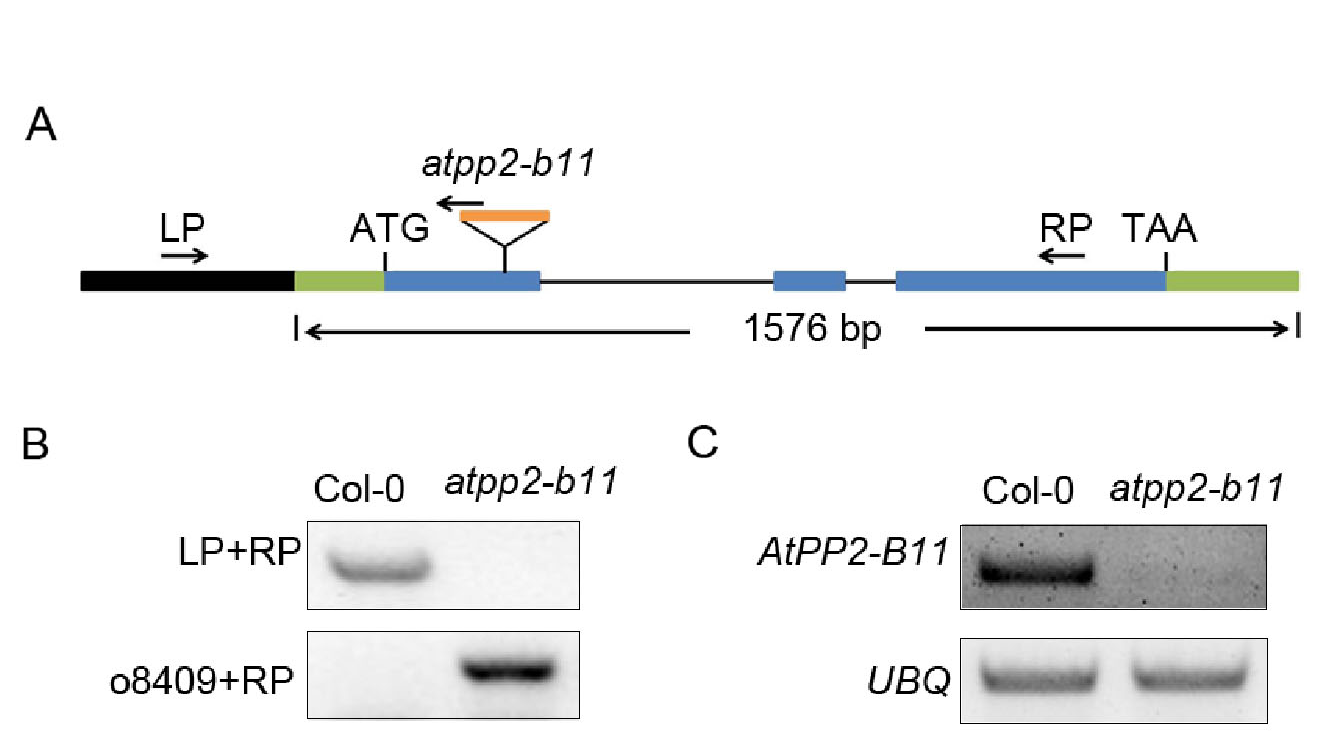

Supplement: S9 Fig — (A). The genomic structure and T-DNA insertions in AtPP2-B11. Exons are depicted as blue boxes, introns are represented by black lines, and 5’and 3’UTR are represented by green boxes, black box represents the promoter region and the black triangle represents the T-DNA insertion site. The arrows represent the primers sites. (B). Identification of homozygous of atpp2-b11. (C). Transcription assay of AtPP2-B11 in Col-0 and atpp2-b11 mutant. (TIF) [file pgen.1006947.s009.tif]

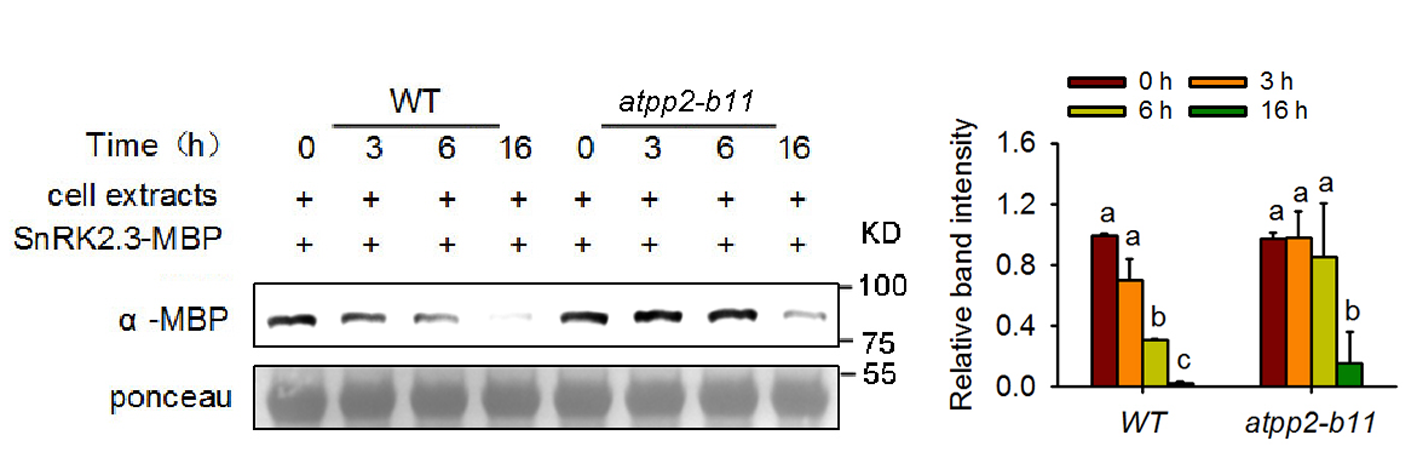

Supplement: S10 Fig — Cell-free assays of SnRK2.3-MBP degradation by incubation of SnRK2.3-MBP with ABA pre-treatment protein extracts from WT or atpp2-b11 knockout mutant. Ponceau staining was used as loading control. Relative amounts of proteins were determined by ImageJ and normalized to loadings determined by Ponceau staining and expressed relative to the value at 0 hr time. Different letters indicate a significant difference (Student-Newman–Kuels [SNK] test, P < 0.05). Quantitative analysis of the band intensity was on the right side of the figure. Error bars are means ± s.e.m. (n ≥ 3 independent experiments). (TIF) [file pgen.1006947.s010.tif]

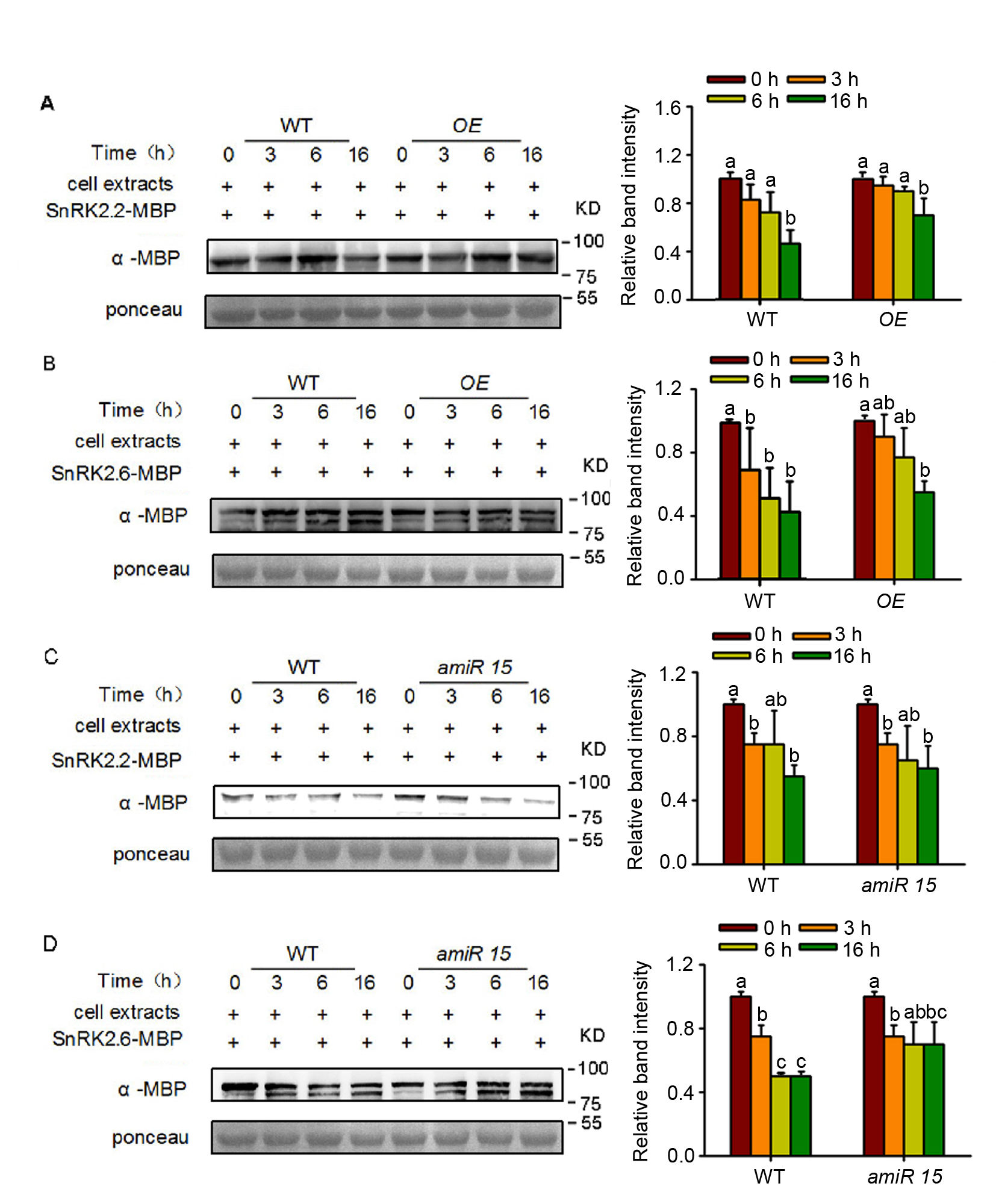

Supplement: S11 Fig — (A). and (B). Cell free degradation of SnRK2.2 and SnRK2.6. Proteins were extracted from 7-day-old seedlings of wild type or AtPP2-B11 overexpression transgenic lines. Ponceau staining was used as loading control. Relative amounts of proteins were determined by ImageJ and normalized to loadings determined by Ponceau staining and expressed relative to the value at 0 hr time. Different letters indicate a significant difference (Student-Newman–Kuels [SNK] test, P < 0.05). Quantitative analysis of the band intensity was on the right side of the figure. Error bars are means ± s.e.m. (n ≥ 3 independent experiments). (C). and (D). Cell free degradation of SnRK2.2 and SnRK2.6. Proteins were extracted from 7-day-old seedlings of wild type or AtPP2-B11 amiRNA knock down line (amiR15). The amiR15 and wild type seedlings were pre-treatment with 50 μM ABA for 5 h. Ponceau staining was used as loading control. Proteins were detected as in (A and B). Different letters indicate a significant difference (Student-Newman–Kuels [SNK] test, P < 0.05). Quantitative analysis of the band intensity was on the right side of the figure. Error bars are means ± s.e.m. (n ≥ 3 independent experiments). (TIF) [file pgen.1006947.s011.tif]

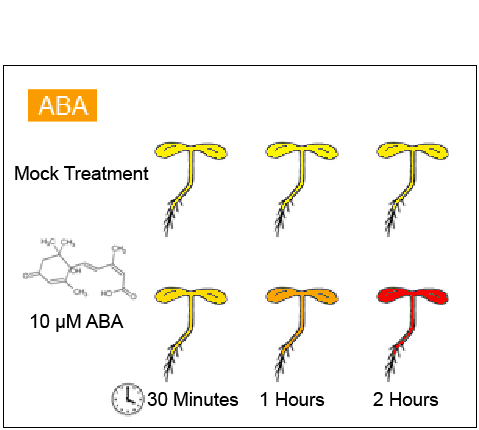

Supplement: S12 Fig — The expression pattern of AtPP2-B11 in response to ABA from the microarray data of public available source (TAIR). (TIF) [file pgen.1006947.s012.tif]

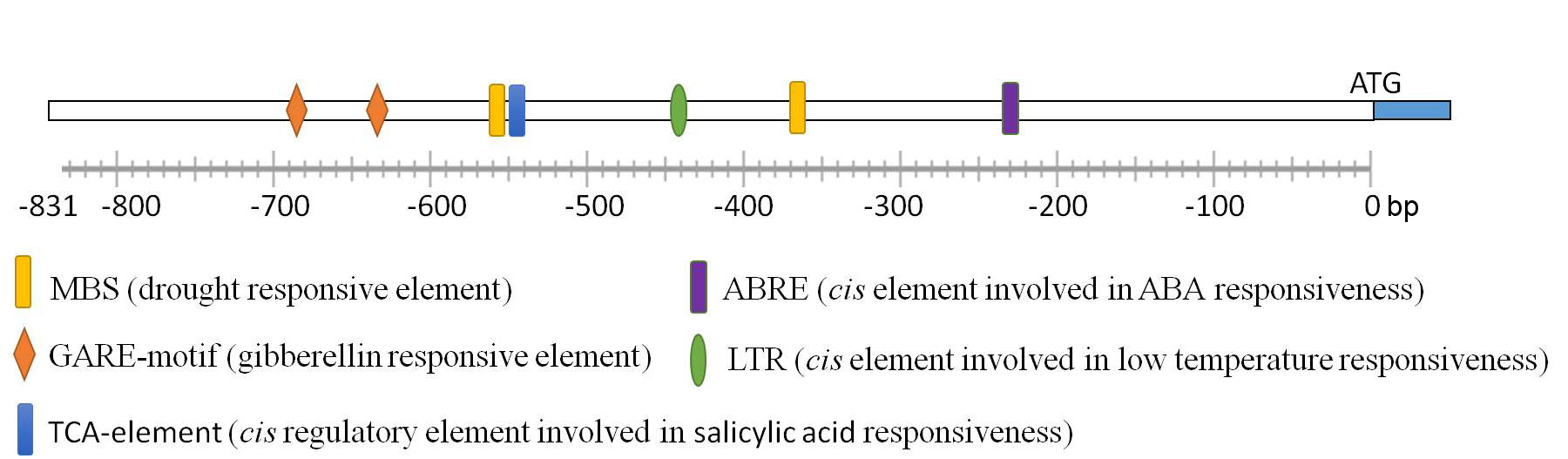

Supplement: S13 Fig — The 831bp DNA fragment upstream of the ATG staring code of the AtPP2-B11 was analyzed using PlantCARE (http://bioinformatics.psb.ugent.be/webtools/plantcare/html/). (TIF) [file pgen.1006947.s013.tif]

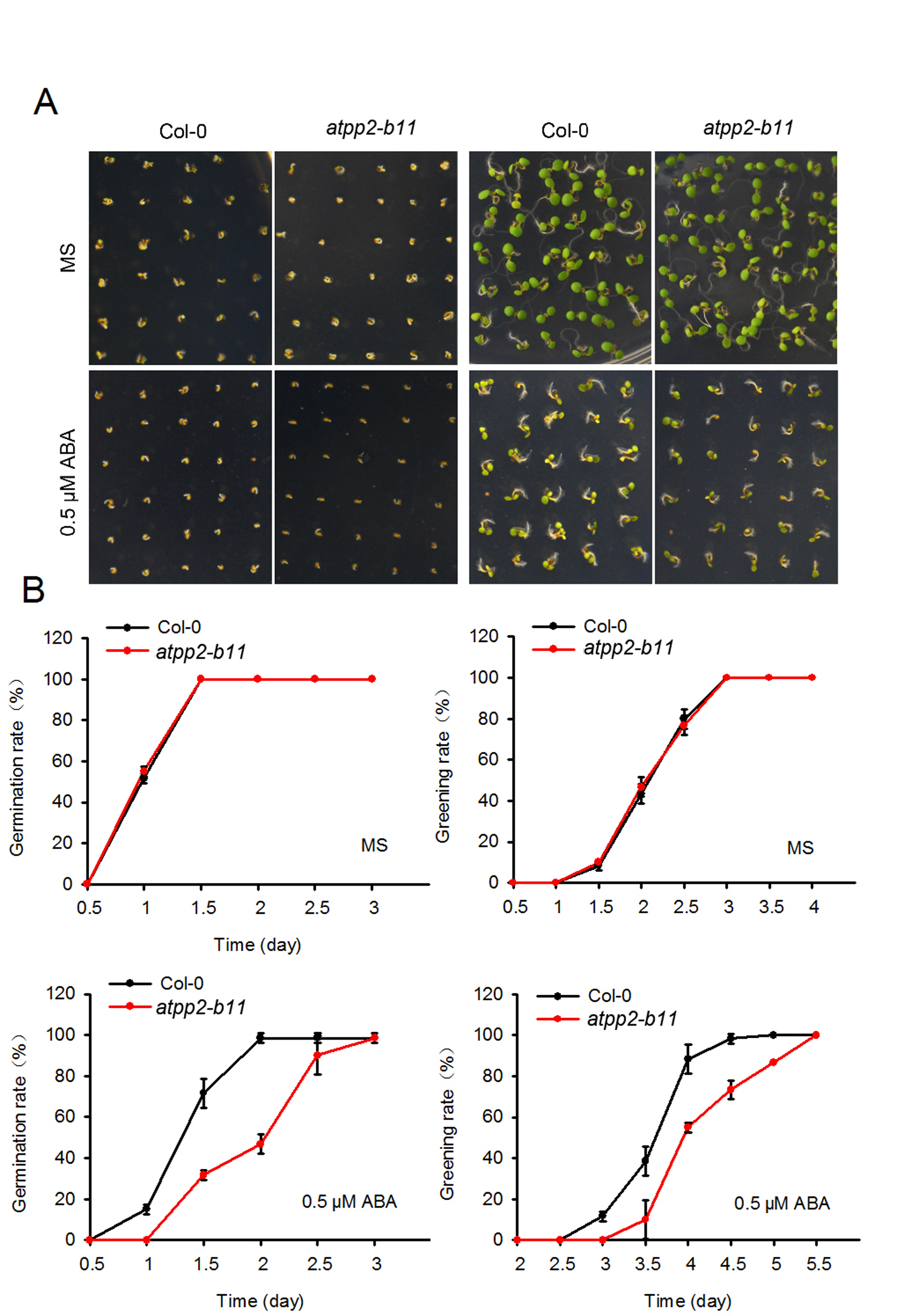

Supplement: S14 Fig — (A) Phenotypic analyses of wild-type (Col-0) and atpp2-b11 treated with 0.5 μM ABA. The images were taken after 4 and 8 days, respectively. (B) The analysis of germination rate and greening rate. (TIF) [file pgen.1006947.s014.tif]

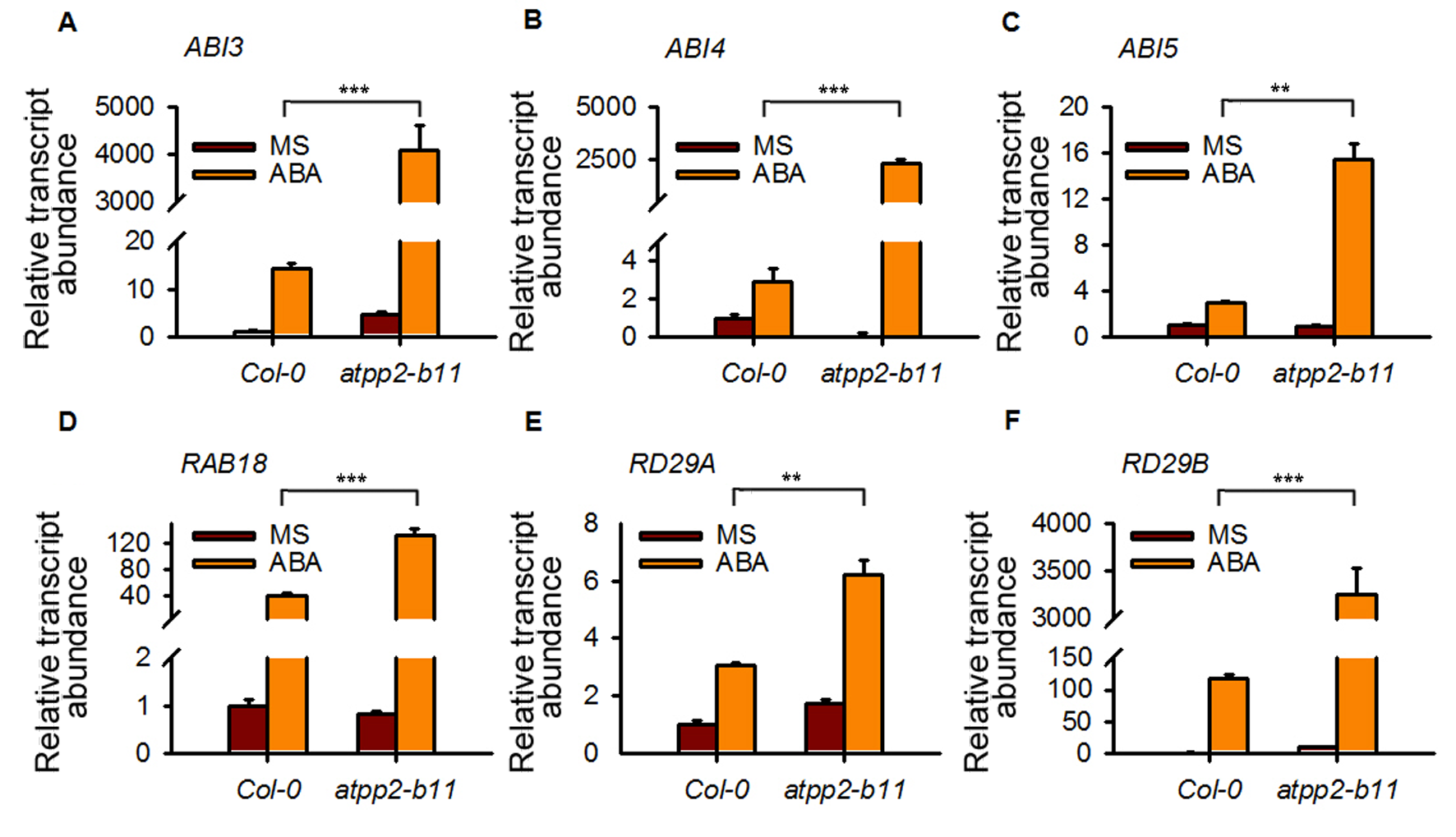

Supplement: S15 Fig — The relative transcript abundance of (A) ABI3, (B) ABI4, (C) ABI5, (D) RAB18, (E) RD29A, and (F) RD29B in Col-0 and atpp2-b11 mutant plants were analyzed. Seedlings were grown on MS medium with or without 0.5 μM ABA for 7 days. Three independent experiments were performed with similar results, each with three replicates. UBC5 was used as the internal control. The student’s t-test was performed and the statically significant treatments were marked with ‘***’ (P < 0.001) and ‘**’ (P < 0.01). (TIF) [file pgen.1006947.s015.tif]
