# Supplementary material for: SCFAtPP2-B11 modulates ABA signaling by facilitating SnRK2.3 degradation in Arabidopsis thaliana
Source: PLoS Genet. 2017 Aug 7;13(8):e1006947. doi: 10.1371/journal.pgen.1006947 (PMC5560758; doi:10.1371/journal.pgen.1006947)
Supplement: S1 Table — (PDF) [file pgen.1006947.s016.pdf]

S1 Table The primers used in this study

Name	DNA sequence(5'-3')	Purpose
SnRK2.2 CDS GWF	GGGGACAAGTTTGTACAAAAAAGCAGGCTTC ATGGATCCGGCGACTAATTC	Y2H; BiFC; Co-IP
SnRK2.2 CDS GWR	GGGGACCACTTTGTACAAGAAAGCTGGGTC CAGAGAGCATAAACTATCTCTCC	Y2H; BiFC; Co-IP
SnRK2.3 CDS GWF	GGGGACAAGTTTGTACAAAAAAGCAGGCTTC ATGGATCGAGCTCCGGTGAC	Y2H; BiFC; Co-IP
SnRK2.3 CDS GWR	GGGGACCACTTTGTACAAGAAAGCTGGGTC GAGAGCGTAAACTATCTCTCCG	Y2H; BiFC; Co-IP
SnRK2.6 CDS GWF	GGGGACAAGTTTGTACAAAAAAGCAGGCTTC ATGGATCGACCAGCAGTGAGTGGTC	Y2H; BiFC; Co-IP
SnRK2.6 CDS GWR	GGGGACCACTTTGTACAAGAAAGCTGGGTC CATTGCGTACACAATCTCTCCGCT	Y2H; BiFC; Co-IP
AtPP2-B11 CDS GWF	GGGGACAAGTTTGTACAAAAAAGCAGGCTTC ATGAATAATCTCCCAGAAGATTG	Y2H; BiFC; Co-IP
AtPP2-B11 CDS GWR	GGGGACCACTTTGTACAAGAAAGCTGGGTC GGGCACTGGTCTAATCTCG	Y2H; BiFC; Co-IP
ASK1 CDS GWF	GGGGACAAGTTTGTACAAAAAAGCAGGCTTC ATGTCTGCGAAGAAGATTGTGTTGA	Y2H; BiFC
ASK1 CDS GWR	GGGGACCACTTTGTACAAGAAAGCTGGGTC TTCAAAAGCCCATTGGTTCTCTCTG	Y2H; BiFC
ASK2 CDS GWF	GGGGACAAGTTTGTACAAAAAAGCAGGCTTC ATGTCGACGGTGAGAAAAATCACTC	Y2H; BiFC
ASK2 CDS GWR	GGGGACCACTTTGTACAAGAAAGCTGGGTC TTCAAACGCCCACTGATTCTCACGG	Y2H; BiFC
AtPP2-B11 Promoter F	CGGGATCCAATTAGAATTTTGTCATAAAAATCA	GUS staining
AtPP2-B11 Promoter R	CGGAATTCTGCTTCCTCCGATTGATTGCTTATT	GUS staining
AtPP2-B11 qRT F	GTTTATCGGCGGTCTCCAGT	qRT PCR
AtPP2-B11 qRT R	TTGCTTCCTGGTGGGAAGAC	qRT PCR
ACTIN2 qRT F	GCCATCCAAGCTGTTCTCTC	qRT PCR
ACTIN2 qRT R	GCTCGTAGTCAACAGCAACAA	qRT PCR
ABI3 qRT F	CACAGCCAGAGTTCCTTCCTTTACT	qRT PCR
ABI3 qRT R	TAGTTGCTGAGGAACACAAACGG	qRT PCR
ABI4 qRT F	GGGCAGGAACAAGGAGGAAGTG	qRT PCR
ABI4 qRT R	TCTCCTCCAAAAGGCCAAATGGT	qRT PCR
ABI5 qRT F	ATGATCAAGAACCGCGAGTCTGC	qRT PCR
ABI5 qRT R	CGGTTGTGCCCTTGACTTCAAAC	qRT PCR
RAB18 qRT F	GGC TTG GGA GGA ATG CTT CA	qRT PCR
RAB18 qRT R	CGC TTG AGC TTG ACC AGA CT	qRT PCR
RD29A qRT F	GGAAGTGAAAGGAGGAGGAGGAA	qRT PCR
RD29A qRT R	CACCACCAAACCAGCCAGATG	qRT PCR
RD29B qRT F	GAATCAAAAGCTGGGATGGA	qRT PCR
RD29B qRT R	TGCTCTGTGTAGGTGCTTGG	qRT PCR

SnRK2.2 CDS F	CCGGAATTCATGGATCCGGCGACTAATTC	Cell free; pull down
SnRK2.2 CDS R	ACGCGTCGACTCAGAGAGCATAAACTATCTCTCCA	Cell free; pull down
SnRK2.3 CDS F	CCGGAATTC ATGGATCGAGCTCCGGTGACC	Cell free; pull down
SnRK2.3 CDS R	CGCGGATCCTTAGAGAGCGTAAACTATCTCTCCG	Cell free; pull down
SnRK2.6 CDS F	CCGGAATTC ATGGATCGACCAGCAGTGAGTGGTC	Cell free; pull down
SnRK2.6 CDS R	CGCGGATCCCATTGCGTACACAATCTCTCCGCT	Cell free; pull down
AtPP2-B11 CDS F	CCGGAATTCATGAATAATCTCCCAGAAGATTG	Cell free; pull down
AtPP2-B11 CDS R	CCGCTCGAGTTAGGGCACTGGTCTAATCTC	Cell free; pull down
LP	CCAGTGCCAGTATCTTTGCTC	Identification of
RP	TAAAGAGAAGGGGACAGGTGG	homozygous
o8409	ATATTGACCATCATACTCATTGC	and heterozygous of
		atpp2-b11
